# The Bacteriophage vB_CbrM_HP1 Protects Crucian Carp Against *Citrobacter braakii* Infection

**DOI:** 10.3389/fvets.2022.888561

**Published:** 2022-05-06

**Authors:** Chunzheng Huang, Chao Feng, Xiao Liu, Rihong Zhao, Zijing Wang, Hengyu Xi, Hongda Ou, Wenyu Han, Zhimin Guo, Jingmin Gu, Lei Zhang

**Affiliations:** ^1^State Key Laboratory for Zoonotic Diseases, Key Laboratory of Zoonosis Research, Ministry of Education, College of Veterinary Medicine, Jilin University, Changchun, China; ^2^College of Animal Science and Technology, Jilin Agricultural University, Changchun, China; ^3^Department of Clinical Laboratory, The First Hospital of Jilin University, Changchun, China; ^4^Jiangsu Co-Innovation Center for the Prevention and Control of Important Animal Infectious Diseases and Zoonoses, Yangzhou University, Yangzhou, China

**Keywords:** *Citrobacter braakii*, phage, characteristics, genome, antibacterial effect, crucian carp

## Abstract

*Citrobacter braakii* is an opportunistic pathogen that induces aquatic infections in fish and turtles. In this study, a bacteriophage that infects *C. braakii*, named vB_CbrM_HP1, was isolated from sewage. This phage belongs to *Myoviridae* family, *Ounavirinae* subfamily, Mooglevirus genus. We also used the phage to treat crucian carp infection caused by *C. braakii* for the first time. vB_CbrM_HP1 was relatively stable at temperatures ranging from 4 to 60°C and pH values ranging from 3 to 11 but float slightly. When the multiplicities of infection (MOI) was 0.0001, the titer reached a maximum of 4.20 × 10^10^ PFU/ml. As revealed from the results of whole genomic sequence analysis, the total length of vB_CbrM_HP1 was 89335 bp, encoding 135 ORFs, 9 of which were <75% similar to the known sequences in NCBI. The phage vB_CbrM_HP1 showed a highly efficient bactericidal effect against *C. braakii* both *in vitro* and *in vivo*. *In vitro*, vB_CbrM_HP1 was capable of effectively killing bacteria (the colony count decreased by 4.7 log units at 5 h). *In vivo*, administration of vB_CbrM_HP1 (1 × 10^9^ PFU) effectively protected crucian carp against fatal infection caused by *C. braakii*. Phage treatment reduced the levels of inflammatory factors. All these results demonstrated the potential of vB_CbrM_HP1 as an alternative treatment strategy for infections caused by *C. braakii*.

## Introduction

Aquaculture products are not only an important part of the human diet but also an important source of protein for human intake (1). The genus *Citrobacter* (*Citrobacter spp*.) is a Gram-negative bacillus genus and comprises a distinct group of 15 species of opportunistic pathogens in the intestinal tract: *Citrobacter amalonaticus, Citrobacter braakii, Citrobacter portucalensis, Citrobacter freundii, Citrobacter rodentium, and Citrobacter koseri*, etc (2). They belong to the *Enterobacteriaceae* family and exist in sewage, water bodies, the human gut, and animal intestines (3). Among them, *C. braakii, C. freundii*, and *C. koseri* are common pathogens causing aquatic infection (4). Compared with *C. freundii* and *C. koseri*, there have been few related studies on *C. braakii* (5). In addition to infecting aquatic animals, *C. braakii* can also cause bacteraemia, neonatal meningitis, brain abscess, intestinal and urinary tract infection in humans and other animals (6, 7). Antimicrobial resistance (AMR) is a global health concern, and its severity is gradually increasing (8, 9). At present, antibiotics are mainly used to treat *C. braakii* infections (10). However, the beta-lactamase gene *blaKPC-2* and the plasmid-mediated colistin resistance gene *mcr-1* have been detected in *C. braakii* (11, 12). Compared with the traditional method (chemical drug therapy) to treat fish diseases, some rising biocontrol methods have attracted the attention of scientists, such as probiotics, bioencapsulated vaccines and phage therapy (1, 13, 14).

Bacteriophage (phage) is a type of bacterial virus with special recognition ability (15). Lytic phages can kill bacteria at the end of the phage infection cycle (16). Phage therapy has an outstanding advantage in preventing and controlling fish diseases, because there is no drug residue (14). A large number of successful cases of phage treatment of aquatic organisms have been reported. Phage VP-2 can effectively reduce the mortality of zebrafish larvae infected with *V. anguillarum* (14). Phage FCP1 can significantly reduce the bacterial population of *F. columnare* in the serum, gills, liver, and kidney of infected fish (17). Using a low-dose (10^2^ PFU/ml) of phage vB_VhaS-tm to treat abalone-infected *V. harveyi*, the survival rate of abalone can be increased by 70% (18). Phage IME-JL8 can effectively prevent and treat carp *Citrobacter freundii* infection. When the fish were treated with phage IME-JL8 at 1 and 12 h after *Citrobacter freundii* infection, the bacterial population decreased at 6 h after infection and decreased to 3.3–3.7 log units at 18 h after infection (19).

At present, there are few studies on *Citrobacter* phages, and they mainly focus on their characterization and genome sequencing analysis. There are approximately 40 *Citrobacter* phages published in PubMed. Only five *Citrobacter* phages (*C. freundii* phage IME-JL8, LK1, phiCFP-1, CfP1, and HCF1) have been characterized thus far (19–23). *C. freundii* phage IME-JL8 was used to study the antimicrobial activity in carp both *in vivo* and *in vitro* (19). *Citrophage* MRM19 and *Citrophage* MRM57 were made into cocktails for an *in vivo* study in a zebrafish model (Danio rerio) (24).

In this study, vB_CbrM_HP1 was isolated from *C. braakii* for the first time. The characterization and whole genome of vB_CbrM_HP1 are presented. According to the sequencing and genome analysis, the phage lacks ORFs related to bacterial toxicity or lysogeny, so the phage is suitable for phage therapy. Moreover, the phage showed good antibacterial effects both *in vivo* and *in vitro*. Thus, phage therapy may be an effective method and shows high potential in the treatment of *C. braakii*-related diseases.

## Materials and Methods

### Ethics Statement

All animal experimental procedures were performed rigorously in compliance with the Regulations for the Administration of Affairs Concerning Experimental Animals, as approved by the State Council of the People's Republic of China (1988.11.1) and the Animal Welfare and Research Ethics Committee at Jilin Agriculture University (JLAU08201409).

### Animal Feeding

In this study, specific pathogen-free and clinically healthy common carp (average weight 50 ± 1 g) specimens were provided by a commercial fish farm and employed for subsequent studies. Fish were maintained in 200 L flow-through tanks at 25 ± 1°C under a natural photoperiod. Fish were fed a commercial diet twice a day at a feeding rate of 1% body weight.

### Bacterial Strains and Growth Conditions

The strains used in this study were stored in our laboratory and are listed in Supplementary Table S1. 8 *Escherichia coli* strains, 8 *Salmonella* strains, 28 *Citrobacter freundii* strains and 6 *Citrobacter braakii* strains were used in this study. The *E. coli* strains, *Salmonella* strains and 16 isolates of *C. freundii* were isolated from the swine samples. The other 12 isolates of *C. freundii* and Cbr-1 were collected from the crap samples. 5 *C. braakii* strains were provided by Professor Xiaofeng Shan. All the strains were stored in liquid medium containing 30% glycerol at −80°C and subsequently cultured in Lysogeny broth (LB) (10 g/L tryptone, 10 g/L NaCl, and 5.0 g/L yeast extract) at 37°C with 180 rpm or on plates (10 g/L tryptone, 10 g/L NaCl, and 5.0 g/L yeast extract 15g/L agar) at 37°C overnight.

### Isolation, Purification, and Host Range of the *C. braakii* Phage vB_CbrM_HP1

Cbr-1 served as a host to isolate phages from five sewage samples (Changchun, Jilin, China). The method of phage isolation was performed as described previously with modifications (25, 26). In brief, 1 ml of Cbr-1 strain was added to 100 ml of sewage (filtered with four-layer gauze), and cocultured in LB medium at 37°C with 180 rpm overnight. The next day, the coculture was centrifuged for 5 min (10,000 × g, 4° C) and the supernatant was filtered using a sterile filter with a pore size of 0.22 μM (Millex-GP Filter Unit; Millipore, Bedford, MA, USA; LOT R6MA05262). A spot test was used to verify the presence of the phage (26). The phage was purified by the double-layer agar plate method (27) as described previously until a uniform plaque was formed (25, 28). In brief, the filtered phage supernatant and host strain cbr-1 were added to 5 ml LB medium for coculturing at 37°C with 180 rpm until the liquid became clear. After the supernatant was filtered by the above method, the host strain Cbr-1 and the supernatant were incubated for 5 min, and then the plaque was cultured by double-layer plate method, and then a single phage was selected to amplify the phage. The cycle was then repeated until the plaques were homogeneous. The purified phage vB_CbrM_HP1 was stored at 4°C and −80°C in 30% glycerol.

In this study, the host range of vB_CbrM_HP1 was determined by the spot test method as described previously (25, 29).

### Biological Characteristics of vB_CbrM_HP1

The multiplicity of infection (MOI) is the ratio of phage titer to the number of host bacteria (30). A slight modification was made on the basis of a previously described method (31). In short, the host strain Cbr-1 was cultured in LB to a logarithmic growth stage (OD_600_ nm = 0.6), centrifuged at 10,000 × g for 5 min at 4°C, washed with the same amount of PBS three times, and suspended with PBS. The phage and host bacteria (10^8^ CFU/ml) were added to LB broth medium at different MOIs (0.0000001, 0.000001, 0.00001, 0.0001, 0.001, 0.01, 0.1, 1 or 10) and incubated at 37°C for 7 h (180 rpm). The phage titer was measured by the double-layer agar plate method (27). This experiment was repeated in triplicate.

The one-step growth curve of the phage was performed on the basis of a previously described method with modifications (25, 31, 32). In short, Cbr-1 was cultured to the logarithmic growth stage (OD_600_ nm = 0.6, 10^8^ CFU/ml) and adsorbed by phage with a MOI of 0.1 at 37°C for 5 min and then centrifuged at 10,000 × g for 15 min at 4°C. Then, the pellet was resuspended in 10 ml of fresh LB broth and cultivated at 37°C with shaking at 180 rpm for 2 h. The titer was determined by the double-layer agar plate method (27). This experiment was repeated in triplicate.

The pH and temperature stability tests were performed on the basis of a previously described method (33). vB_CbrM_HP1 with the same titer (10^10^ PFU/ml) was incubated in SM buffer (pH 2–11) at 37°C for 2 h. For the temperature stability test, vB_CbrM_HP1 with the same titer (10^10^ PFU/ml) was incubated at 4°C, 25°C, 37°C, 50°C, 60°C, 70°C and 80°C, and samples were taken every 20 mins to determine the phage titer. In the above two experiments, the phage titer was determined by the double-layer agar plate method (27)and repeated in triplicate.

### Phage Morphology and Nucleic Acid Type Identification

The morphology of vB_CbrM_HP1 was observed by transmission electron microscopy (TEM) (JEOL JEM-1200EXII, Japan Electronics and Optics Laboratory, Tokyo, Japan). Concentrated vB_CbrM_HP1 samples were placed on carbon-coated copper grids to absorb for 15 min and then were negatively stained with phosphotungstic acid (PTA, 2% w/v). The morphology of vB_CbrM_HP1 was examined using TEM at an acceleration voltage of 80 kV (34).

With the UNIQ-10 Column Virus Genomic DNA Isolation Kit (Sangon, Shanghai, China), phage genomic DNA was extracted and stored at −80°C for sequencing. Phage nucleic acids were digested with DNase I (10.0 U/μg), RNase A (10.0 U/μg) and mung bean nucleus (20.0 U/μg) for 1 h. The nucleic acid type of the digested samples was analyzed by agarose gel electrophoresis.

### vB_CbrM_HP1 Genome Sequencing and Analysis

The whole genome of vB_CbrM_HP1 was sequenced using Illumina NovaSeq PE150. The process of the Library construction is generally as follows. In general, the genomic DNA was extracted with the SDS method (35). The harvested DNA was detected by the agarose gel electrophoresis and quantified by Qubit® 2.0 Fluorometer (Thermo Scientific). A total amount of 1 μg DNA per sample was used as input material for the DNA sample preparations. Sequencing libraries were generated using NEBNext® Ultra™ DNA Library Prep Kit for Illumina (NEB, USA) following manufacturer's recommendations and index codes were added to attribute sequences to each sample. Briefly, the DNA sample was fragmented by sonication to a size of 350 bp, then DNA fragments were end-polished, A-tailed, and ligated with the full-length adaptor for Illumina sequencing with further PCR amplification. At last, PCR products were purified (AMPure XP system) and libraries were analyzed for size distribution by Agilent2100 Bioanalyzer and quantified using real-time PCR. The SOAP denovo, SPAdes, Abyss, CISA gapclose software were used to assemble the sequences.

Genome annotation and potential open reading frames (ORFs) were initially annotated with Rapid Annotation using Subsystem Technology [RAST (https://rast.nmpdr.org/)] and then further analyzed by using blast and HMMER (https://www.ebi.ac.uk/Tools/hmmer/search/phmmer). A tRNA scanner (http://lowelab.ucsc.edu/tRNAscanSE/index.html) was used to analyse tRNA. A schematic diagram of the gene function module map was drawn with CLC main workbench version 7.7.3 software (CLC bio Qiagen, Aarhus, Denmark). Amino acid sequences of the terminase large subunits and lysome of vB_CbrM_HP1 were used to make phylogenetic trees. The Clustalw program within BioEdit 7.0.4.1 was used for packaging. Based on the related gene sequences and the sequence alignment results, the maximum likelihood (ML) phylogenetic trees were constructed using Phylip software (version 3.697) (25).

### Antimicrobial Activity of the Phage vB_CbrM_HP1 *in vitro*

vB_CbrM_HP1 was determined according to a previous method (31). In short, Cbr-1 was cultured in LB broth medium until the logarithmic growth period (OD_600_ nm = 0.6, 10^8^ CFU/ml) and washed three times with PBS. Phage and host bacterium Cbr-1 were added to LB broth medium with different MOI (PFU/CFU) values (0.0000001, 0.00001, 0.001, 0.1, or 10). After culturing at 37°C with shaking at 180 rpm, 50 μl of culture samples were taken every 1 h for 6 h. The concentration of live Cbr-1 was determined by continuous dilution and inoculation on LB agar plates.

### Treatment Effect of Phage vB_CbrM_HP1 *in vivo*

Experimental methods were performed according to the previous experiments with modifications (19, 34, 36). We evaluated phage vB_CbrM_HP1 for the treatment of *C. braakii* infection in crucian carp. We first verified the safety of phage vB_CbrM_HP1 by dividing crucian carp into two groups (*n* = 6 in each group): one group was intraperitoneally injected with phage vB_CbrM_HP1 (1 × 10^9^ PFU/fish), and the other group was intraperitoneally injected with PBS. The minimum lethal dose (MLD) for Cbr-1 was then determined. Six crucian carp in each group were intraperitoneally injected with Cbr-1 at different concentrations (10^6^, 10^7^, 10^8^, 10^9^ and 10^10^ CFU/fish), the control group was injected with PBS. Crucian carp death was recorded within 7 days, and 2 × MLD was used as the dose of Cbr-1 infection.

Crucian carp were infected with 2 × MLD (2 × 10^9^ CFU/fish) of Cbr-1 for 1 h and then intraperitoneally injected with phage vB_CbrM_HP1 (1 × 10^6^, 1 × 10^7^, 1 × 10^8^ and 1 × 10^9^ PFU/fish) (*n* = 20 in each group). To further explore the relationship between the treatment effect of phages *in vivo* and infection time, crucian carp were infected with 2 × MLD (2 × 10^9^ CFU/fish) Cbr-1 and treated with phage vB_CbrM_HP1 (1 × 10^9^ PFU/fish) at 12 and 24 h after infection (*n* = 60 in each group). The control group was given the same amount of PBS (100 μl/fish). The survival rate of crucian carp was recorded over 7 days.

Histopathological analysis of the gut, liver and spleen of the crucian carp that received different treatments was performed. Briefly, phage treatment was performed 1 h, 12 h and 24 h after infection (*n* = 6 in each group), and three crucian carp in each group were euthanized at different time points after treatment. The gut, liver and spleen of the crucian carp were removed and immediately placed in 4% formalin. The formalin-fixed tissues were processed, stained with haematoxylin and eosin, and analyzed by microscopy.

The transcription levels of intestinal cytokines (TNF-α, IFN-γ and IL-1β) were detected by qPCR at 6, 12 and 18 h after phage vB_CbrM_HP1 treatment (n = 6 in each group). Crucian carp treated with PBS were used as the control group. Primers for immune-related genes and β-actin are shown in Supplementary Table S2.

### Statistical Analysis

The statistical data involved in this study were processed by one-way analysis of variance (ANOVA) or Student's t tests. All pictures were generated by GraphPad Prism 8.0 (GraphPad Software, USA). The error bar represents standard deviation of the mean. ^*^P<*0*. *05* indicates a significant difference in the data; ^**^P<*0*. *01*, ^***^P<*0*. *001* indicates a very significant difference in the data.

## Results

### Phage Purification and Characteristics

**Figure 1:**
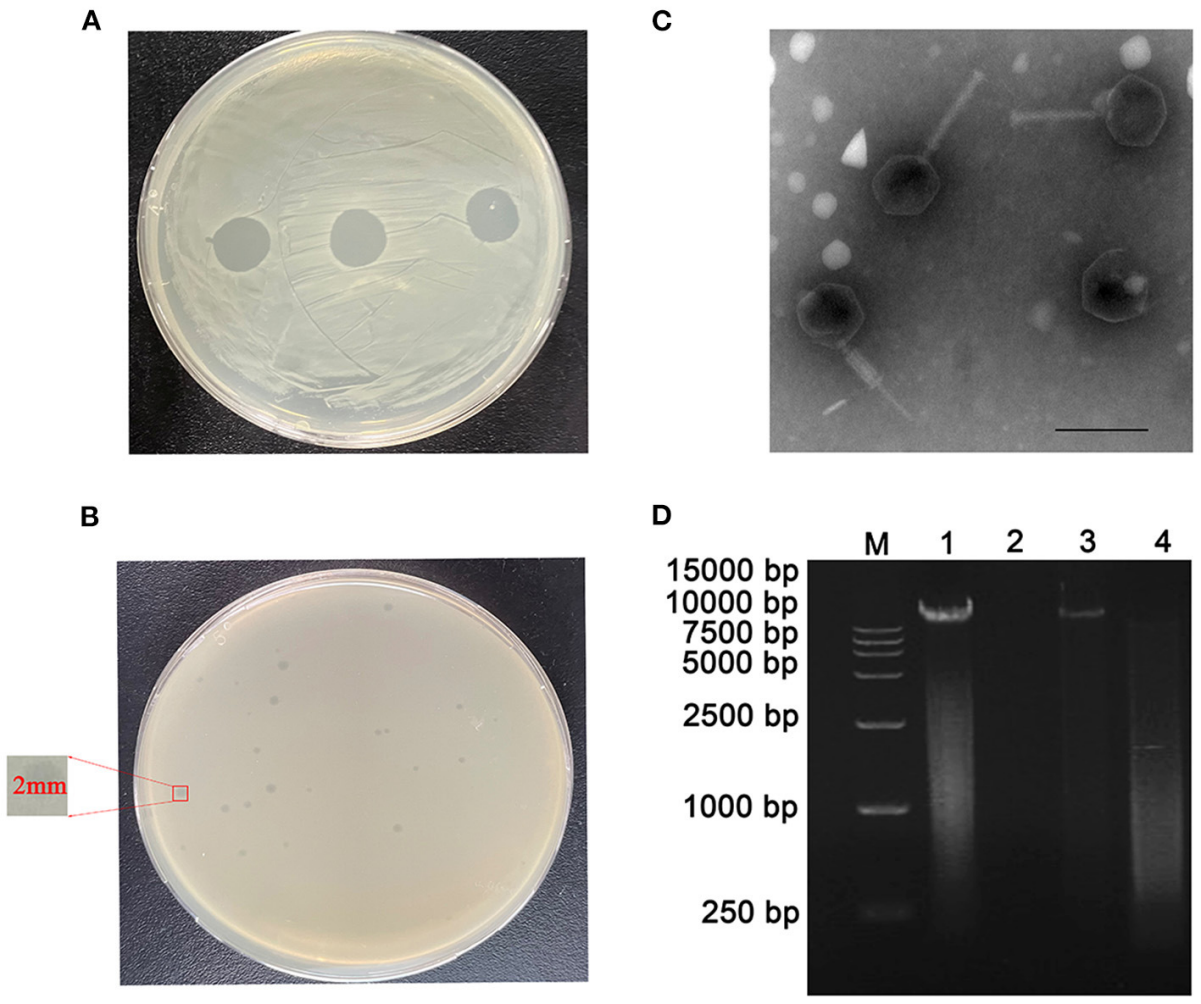
Characteristics of phage vB_CbrM_HP1. **(A)** Spot tests of phage vB_CbrM_HP1. **(B)** Plaque observation of phage vB_CbrM_HP1. Each single plaque was ≈ 2 mm in diameter. **(C)** Transmission electron microscopy (TEM) of phage vB_CbrM_HP1 at an accelerating voltage of 80 kV. Phage vB_CbrM_HP1 negatively stained with 2% phosphotungstic acid (PTA), and the scale bars represent 100 nm. **(D)** Nucleic acid type identification of phage vB_CbrM_HP1. M: 15,000 bp Marker; 1: nucleic acid of phage; 2: DNase I (10.0 U/μg); 3: RNase A (10.0 U/μg); 4: mung bean nuclease (20.0 U/μg).

Using Cbr-1 as the host bacterium, a lytic phage was isolated from sewage and showed a transparent spot in the lawn of Cbr-1 (Figure 1A). It was purified by the double-layer agar plate method and named vB_CbrM_HP1. Phage vB_CbrM_HP1 formed a transparent plaque with a diameter of 2–3 mm (Figure 1B). The electron micrograph of vB_CbrM_HP1 showed that it had an isometric icosahedral head and a contracted tail, which indicated that vB_CbrM_HP1 was a member of the *Myoviridae* family, *Ounavirinae* subfamily, Mooglevirus genus (Figure 1C). This result indicated that the genome of phage vB_CbrM_HP1 was double-stranded DNA (Figure 1D). We selected 8 *Escherichia coli* strains, 8 *Salmonella strains*, 6 *Citrobacter braakii* and 28 *Citrobacter freundii* strains to determine the host range of vB_CbrM_HP1. We found that vB_CbrM_HP1 had a relatively narrow host range. Apart from Cbr-1, vB_CbrM_HP1 could lyse only 3 of the 28 tested *Citrobacter freundii* strains (Supplementary Table S1).

**Figure 2:**
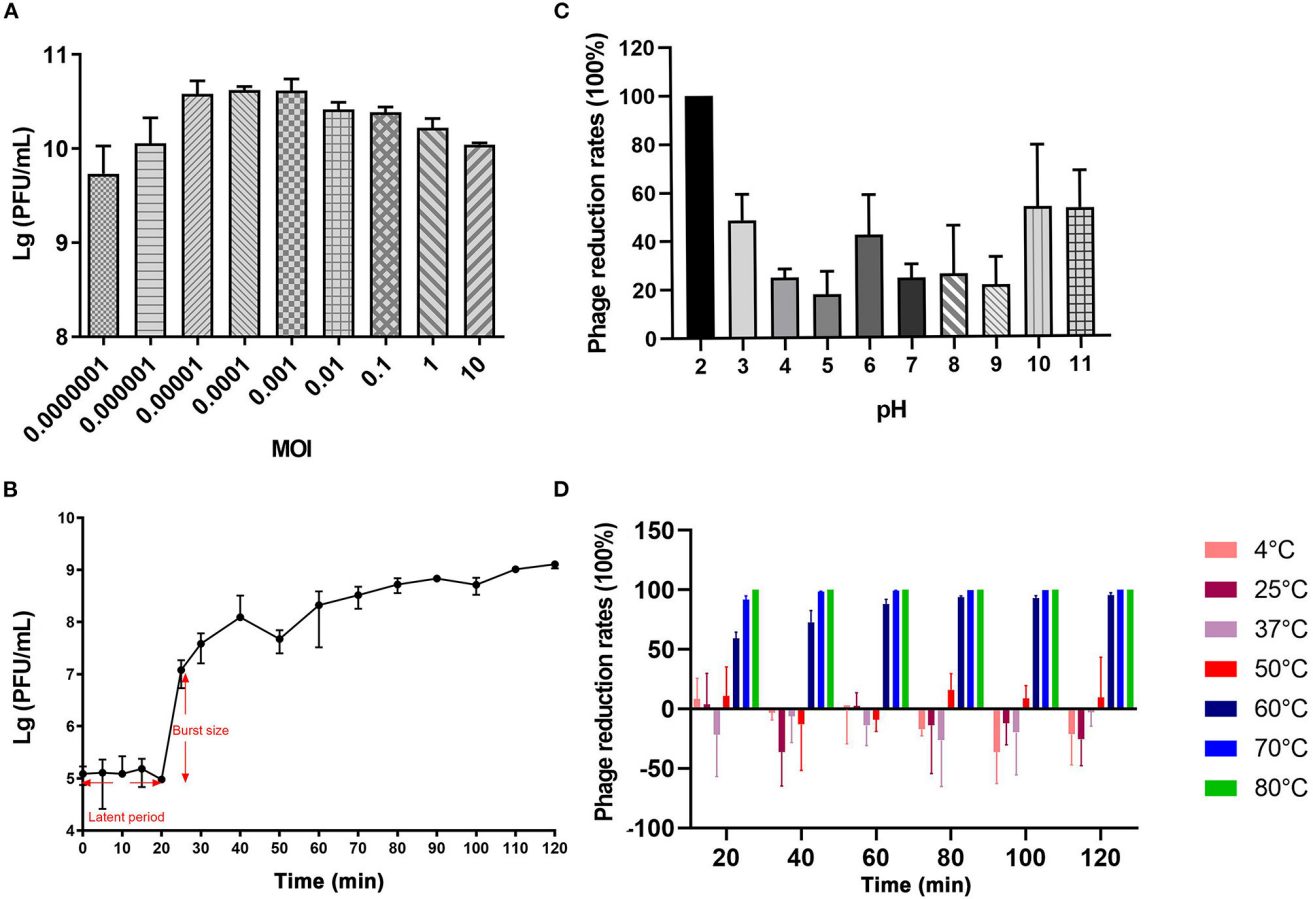
Growth characteristics and stability tests of vB_CbrM_HP1. **(A)** The optimum MOI of phage vB_CbrM_HP1. The titer of vB_CbrM_HP1 at nine different multiplicities of infection (MOIs) at 7 h at 37°C with 180 rpm/min. **(B)** The one-step growth curve of phage vB_CbrM_HP1. When the MOI was 0.1, the titer of vB_CbrM_HP1 changed within 2 h. **(C)** The pH stability of phage vB_CbrM_HP1. The reduction rates of vB_CbrM_HP1 in SM buffer of different pH (2–11) values for 2 h at 37°C. **(D)** The temperature stability of phage vB_CbrM_HP1. The reduction rates of vB_CbrM_HP1 at different temperatures (4–80°C) over 2 h. The means and standard deviations are represented as points with error bars (*n* = 3).

vB_CbrM_HP1 had a smaller optimum MOI, shorter latent period, and shorter outbreak period. The burst size was approximately 126 PFU/cell. The results showed that vB_CbrM_HP1 had high reproduction efficiency and lysis activity. The optimum MOI of vB_CbrM_HP1 was 0.0001, and the titer reached 4.2 × 10^10^ PFU/ml (Figure 2A). The results of the one-step growth curve of vB_CbrM_HP1 showed that the latent phase of Cbr-1 was 20 min, the burst time was approximately 10 min, and a lysis cycle took ~40 min. The burst size was approximately 126 PFU/cell (Figure 2B). The titer and survival rate of vB_CbrM_HP1 was relatively stable at temperatures ranging from 4°C to 60°C and pH values ranging from 3 to 11 but float slightly (Figures 2C,D). vB_CbrM_HP1 maintained a certain survival rate ranging from 3 to 11. At pH 12, the titer of phage decreased by only 50%. When pH <2, the activity of vB_CbrM_HP1 was lost (Figure 2C). Remarkably, the titer of vB_CbrM_HP1 significantly decreased at 70°C and 80°C. It was almost completely inactivated after 20 min of treatment at 80°C. After being treated with 60°C for 120 min, 4.58% of vB_CbrM_HP1 still survived, and the titer remained at 2.23 × 10^4^ (Figure 2D). These indicated that vB_CbrM_HP1 showed excellent temperature and pH stability.

### General Features of the vB_CbrM_HP1 Genome

**Figure 3:**
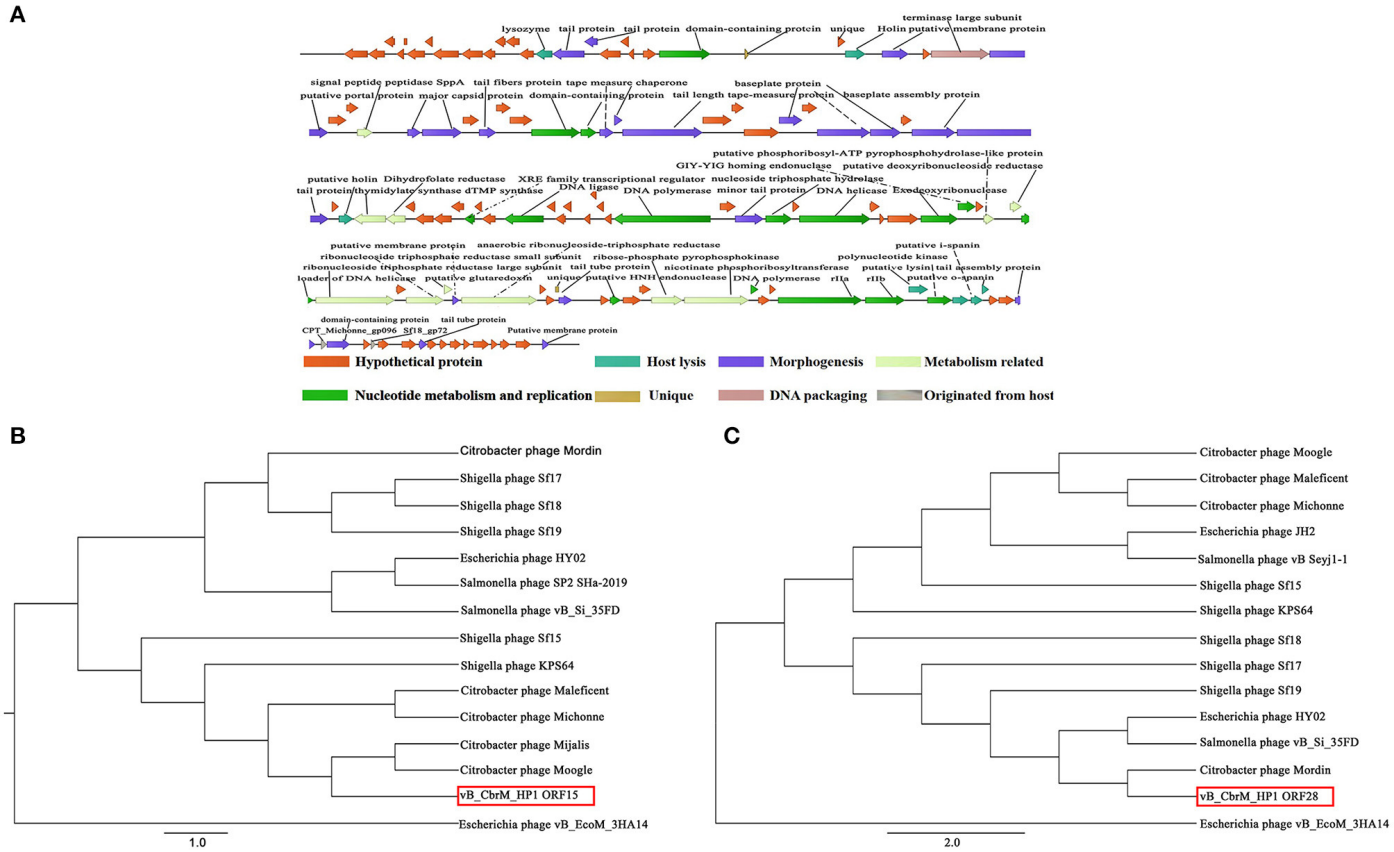
Genome analysis of vB_CbrM_HP1. **(A)** The functional map of phage vB_CbrM_HP1. Each arrow represents an open reading frame (ORF); the direction of the arrow represents the transcription direction, and the functions encoded by the ORFs are annotated above. All encoded proteins were divided into eight modules, and each module is labeled with different colors. **(B)** Phylogenetic tree based on the lysome proteins. **(C)** Phylogenetic tree based on the terminase large subunits. The Clustalw program within BioEdit 7.0.4.1 was used for packaging. Based on the alignment results with the lysome and terminase large subunit gene sequences, the maximum likelihood (ML) phylogenetic tree was constructed using Phylip software (version 3.697).

vB_CbrM_HP1 was sequenced and analyzed. The whole genome sequence of phage vB_CbrM_HP1 has been submitted to GenBank in NCBI (**OK_539913**). CGView was used to construct a map of the phage vB_CbrM_HP1 genome (89,355 bp in length). The content of G + C was 38.90%. The protein sequence encoded by vB_CbrM_HP1 was predicted by BLAST (NCBI). As shown in Supplementary Table S3, 98.52% (133/135) of ORFs used ATG as the start code. A total of 0.74% (1/135) used GTG as the start codon, and 0.74% (1/135) used TTG as the start codon. A total of 63.70% (86/135) of the ORFs used TAA as the stop codon, 9.63% (13/135) used GTG as the stop codon, and 26.67% (36/135) used TTG as the stop codon. vB_CbrM_HP1 had linear genomes with direct repeats at both termini. Twenty-one tRNA genes were identified. The phage vB_CbrM_HP1 genome encodes 135 open reading frames (ORFs). Among them, 64 ORF-encoded proteins had homology with known functional proteins in the NCBI database. Two ORFs (ORF22 and ORF96) were unique proteins of vB_CbrM_HP1. The phage vB_CbrM_HP1 genome was divided into eight modular regions: nucleotide metabolism and replication region, DNA packaging region, morphogenesis region, host lysis region, region originated from the host, hypothetical protein region, metabolism–related protein region, and unique protein region (Figure 3A). The whole genome sequence of vB_CbrM_HP1 was similar to that of *Shigella* phage Sf17 (Query cover, 93%, Identity, 91.44%, **NC_042076.1**), *Citrobacter freundii* phage Mijalis (Query cover, 95%, Identity, 97.73%, **KY654690.2**) and *Escherichia* phage vB_EcoM_AYO145 (Query cover, 74%, Identity, 83.30%, **AKR014248.1**). Although the homology and coverage were very high, the similarity of proteins encoded by ORFs (ORF16, ORF39, ORF70, ORF75, ORF87, ORF92, ORF97, ORF126, and ORF134) was less than 75%. Among them, three of the nine proteins (ORF16, ORF75, and ORF97) might be tail-related proteins. The proteins encoded by ORF22 and ORF96 are unique to vB_CbrM_HP1, and their functions are currently unknown. Moreover, there were another two ORFs encoding putative endolysin (ORF14 and ORF110) and two ORFs encoding holin protein (ORF24 and ORF54). However, no ORFs associated with drug resistance or lysogeny were found. The phylogenetic tree was constructed by the maximum likelihood (ML) method with two relatively conserved genes, including the large terminate subunit gene and lyase gene. The results showed that the lyase gene was closely related to two strains of *C. freundii* phage (Moogle and Mijals) (Figure 3B). However, from the perspective of the terminal large subunit gene, vB_CbrM_HP1 was not closely related to the above two strains of *C. freundii* phage; instead, it was closely related to another *C. freundii* phage (Mordin), *Esherichia* phage HY02 and to *Salmonella* phage (vB_Si_35FD) (Figure 3C).

### Antibacterial Effects of vB_CbrM_HP1 *in vitro*

**Figure 4:**
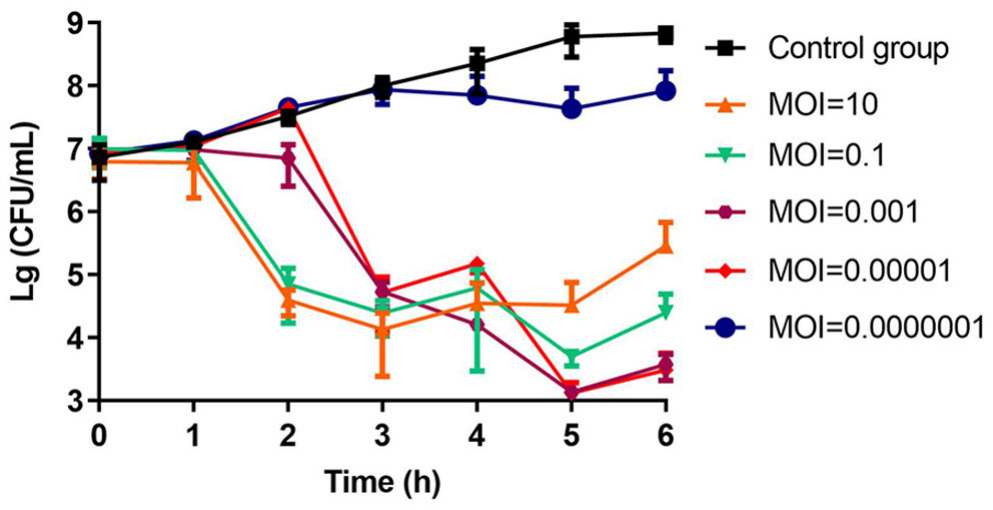
The bactericidal activity of phage vB_CbrM_HP1 *in vitro*. Phage vB_CbrM_HP1 and isolate Cbr-1 were cocultured in LB broth at different MOIs (10, 0.1, 0.001, 0.00001, and 0.0000001). No phage vB_CbrM_HP1 was added to the control group. The changes in the bacterial population in each group over 6 h were measured. The means and standard deviations are represented as points with error bars (*n* = 3).

The number of colonies in the negative control group increased continuously (6 × 10^8^ CFU/ml, 5 h). In contrast, in addition to the group with MOI = 0.0000001, all other phage treatment groups showed significant inhibition of bacterial growth (10^3^-10^5^ CFU/ml, 5 h). When the MOI was 0.001 and 0.00001, the colony decreased most obviously, ~4.7 log units at 5 h. The closer we approached the optimum MOI 0.0001, the more significant the antibacterial effect was. After 5 h, the bacterial population began to increase, indicating that phage-resistant bacteria had proliferated (Figure 4).

### Therapeutic Effect of Phage vB_CbrM_HP1

**Figure 5:**
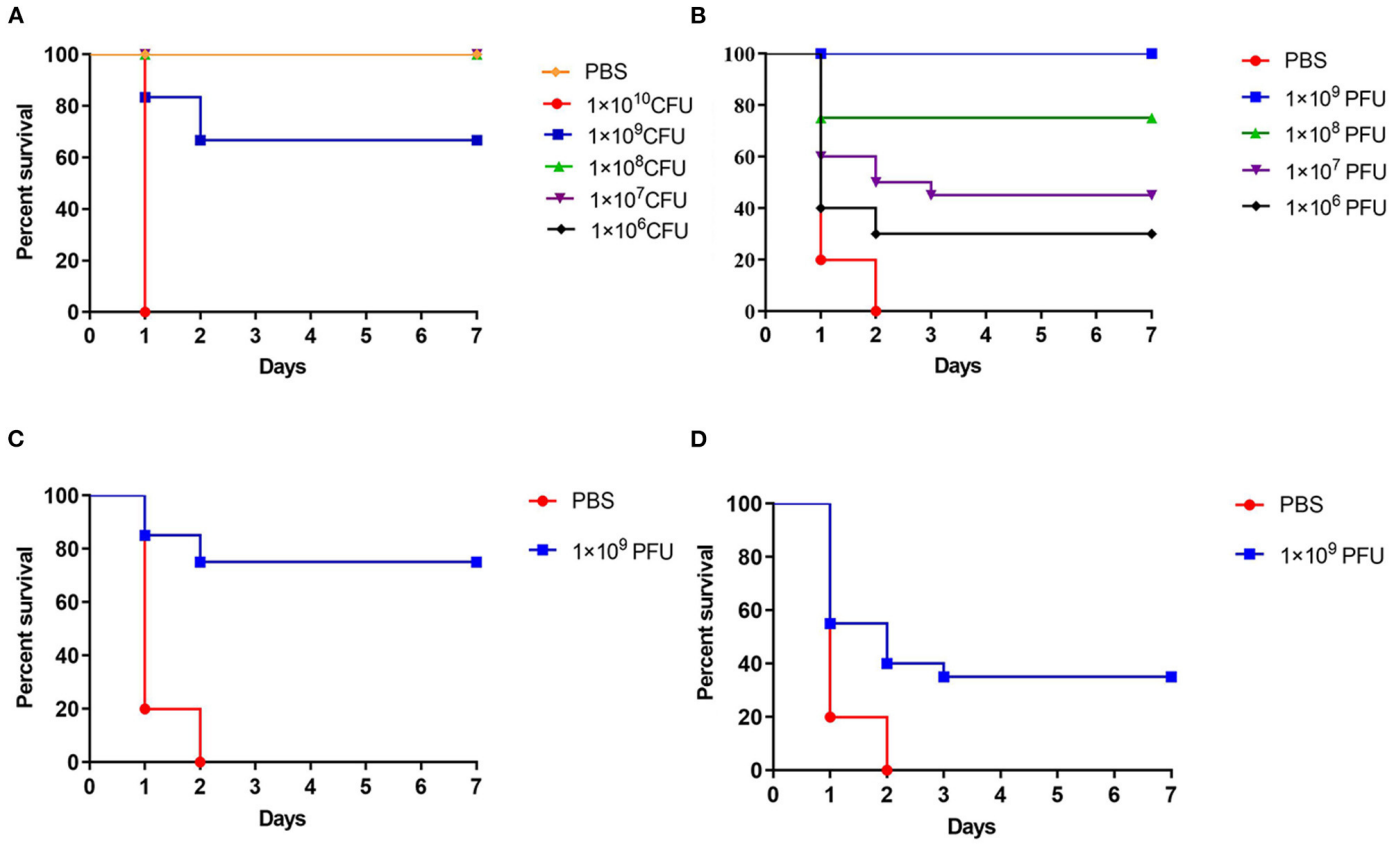
The treatment effect of phage vB_CbrM_HP1 *in vivo*. **(A)** The minimum lethal dose (MLD) of Cbr-1. 10^6^, 10^7^, 10^8^ 10^9^and 10^10^ CFU of Cbr-1 and PBS were introduced intraperitoneally. The fish were intraperitoneally injected with a 2 × minimum lethal dose (MLD) (2 × 10^9^ CFU/fish) of Cbr-1. **(B)** One hour later, 10^6^, 10^7^, 10^8^ and 10^9^ PFU of vB_CbrM_HP1 were introduced intraperitoneally. **(C)** Twelve hours after injection of Cbr-1 (2 × 10^9^ CFU/fish) and **(D)** 24 h after injection of Cbr-1 (2 × 10^9^ CFU/fish), 1 × 10^9^ PFU phage of vB_CbrM_HP1 was introduced intraperitoneally. Control fish were administered PBS under identical conditions. The means and standard deviations are represented as points with error bars.

**Figure 6:**
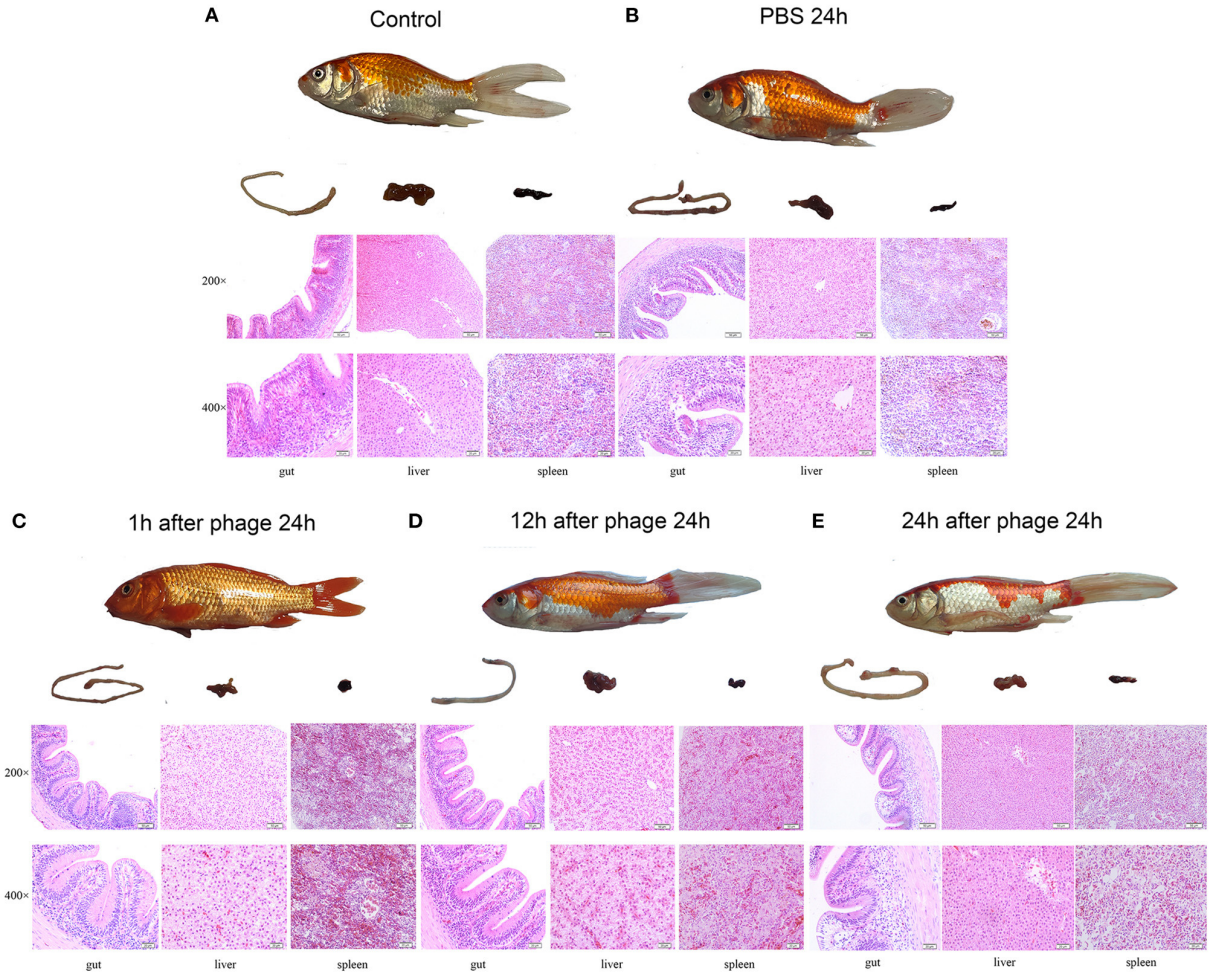
Gross pathology and histopathology. The crucian carp were infected with Cbr-1 (2 × 10^9^ CFU/fish) and then treated with PBS or 1 × 10^9^ PFU vB_CbrM_HP1 at different time points. The gut, liver and spleen tissues were removed from the crucian carp 24 h after treatment with PBS or the phage and then stained with haematoxylin and eosin. **(A)** The gut, liver, and spleen of the healthy crucian carp; **(B)** 1 h after treatment with PBS; **(C)** 1 h after treatment with vB_CbrM_HP1; **(D)** 12 h after treatment with vB_CbrM_HP1; **(E)** 24 h after treatment with vB_CbrM_HP1.

The crucian carp injected with 1 × 10^9^ CFU/ fish, the survival rate was 66.6% after 7 days (Figure 5A). When the crucian carp were infected with Cbr-1 (2 × MLD, 2 × 10^9^ CFU/fish), all crucian carp died within 2 days; in contrast, all carp administered phage vB_CbrM_HP1 (1 × 10^9^ PFU/fish) recovered (Figure 5B). After infection Cbr-1 (2 × 10^9^ CFU/fish) for 12 h and 24 h, 20 surviving crucian carp were injected with phage (1 × 10^9^ PFU/ fish). As shown in Figures 5C,D, phage vB_CbrM_HP1 was sufficient to produce the 75 and 35% survival rate, respectively (Figures 5C,D). Based on histopathological analysis, the normal tissues of the gut, liver and spleen showed intact structures and no inflammatory cell infiltration (Figure 6A). However, *C. braakii* infection caused severe damage to the gut and spleen of crucian carp. In regards to the spleen, 24 h after crucian carp were infected with Cbr-1, spleen bleeding was serious, the cells were arranged loosely and disordered, and some cells showed cytoplasmic vacuolization compared with the normal tissue. In regards to the gut lesions, gut villus shedding and inflammatory cell infiltration were observed (Figure 6B). Compared with the control group of crucian carp, the liver pathological changes in each treatment group were not significant. In contrast, inflammation and pathological changes were significantly alleviated in the gut and spleen tissues from crucian carp that were treated with phage. The gut epithelial villi of crucian carp were unchanged, and the bleeding from the spleen was relieved compared to the control group (Figures 6C–E).

**Figure 7:**
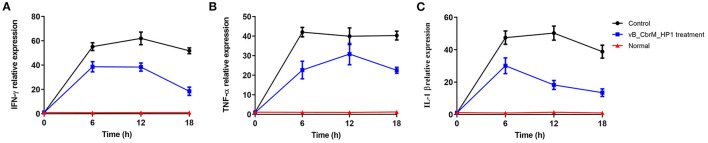
Cytokine levels. The fish were injected intraperitoneally with Cbr-1 (2 × 10^9^ CFU/fish). One hour later, vB_CbrM_HP1 (10^9^ PFU/fish) or PBS was introduced intraperitoneally. **(A)** The transcription levels of the intestinal cytokines IFN-γ, **(B)** TNF-α and **(C)** IL-1β were detected by qPCR at 6, 12 and 18 h after vB_CbrM_HP1 phage treatment. The experiment was repeated three times. Each data point is expressed as the mean ± SD from three biological experiments.

The levels of TNF-α, IL-1β and IFN-γ in the intestine of crucian carp in different treatment groups were detected by qPCR. After 6 h of vB_CbrM_HP1 treatment, the transcription levels of TNF-α, IL-1β and IFN-γ in the intestine showed an upwards trend. Among these cytokines, IFN-γ and IL-1β reached their peaks at 6 h and then appeared to decrease (Figures 7A,C), while TNF-α reached its highest transcription level at the 12th hour (Figure 7B). Compared with the control group, cytokine transcription levels were decreased in all treatment groups and significantly decreased at 18 h (Figure 7).

## Discussion

In recent years, there have been more reports of infections caused by *C. braakii*. *C. braakii* can infect *Chelonia mydas*, a species of sea turtle (37). In addition, this bacterium has also been shown to be the dominant flora causing ulcerative stomatitis lesions and may be related to the pathogenesis of ulcerative stomatitis (37, 38). The literature also showed that *C. braakii* was associated with food contamination, bacteraemia, septicaemia, gastrointestinal infection, neonatal meningitis and brain abscess in humans and animals (7, 39–42). At present, antibiotics are mainly used to treat *C. braakii* infections (10). A report in 2021 showed the high prevalence of MDR in *C. braakii* in China (10). In particular, *C. braakii* was shown to be resistant to carbapenem, and the carbapenemase gene *blaKPC-2* and plasmid-mediated colistin resistance gene *mcr-1* were detected in *C. braakii* (11, 12). Therefore, we need to pay more attention to the control of infections caused by this bacterium. In the face of antibiotic resistance, phage therapy has attracted increasing attention. Compared with antibiotics, phage therapy has many advantages, such as rapid reproduction (43), high specificity, ease of obtaining and safety of its use (44). Phages and their product, phage lysins, can also be mixed with other biological agents to broaden their host range. For example, phages can be combined into phage cocktails with other phages or coupled with antibiotics (45).

In this study, we isolated a phage infecting *C. braakii* from sewage and named it vB_CbrM_HP1. In the PubMed database, approximately 40 published *Citrobacter* phages were found. For most of them, only the complete genome sequence had been uploaded, and no characteristics had been described. For example, from 2015 to 2019, the Kuty Everett GF team uploaded 9 complete genome sequences of *C. freundii* phage (Moogle, Merlin, Maleficent, Maroon, Mijalis, Moon, Michonne, Mordin, and Miller) (46–54), which were similar to vB_CbrM_HP1, but they did not further study their biological characteristics or antibacterial activities *in vivo* or *in vitro*. Only five *Citrobacter* phages (*C. freundii* phage IME-JL8, LK1, phiCFP-1, CfP1, and HCF1) have been characterized thus far (19–23). Research suggested that they were *Siphoviridae* phages (IME-JL8 and HCF1), *Podoviridae* phages (LK1 and phiCFP-1), or a *Myovirus* phage (CfP1). Additionally, as a *Myovirus* phage, CfP1 had a total genome length of 180,219 bp, which was much longer than that of vB_CbrM_HP1 (89355 bp). The latency of phiCFP-1 was almost the shortest at approximately 20 min. The greatest burst size of LK1 was 801 PFU/cell. These phages were relatively stable at temperatures ranging from 4°C to 50°C and pH values ranging from 4 to 10. CfP1 had a broad host range (>85%; 21 strains tested) and specifically infects *C. freundii*. Notably, HCF1 infected both *C. amalonaticus* and *C. freundii*. The *Citrophage* IME-JL8 had good antimicrobial activity.

We studied the biological characteristics and antibacterial effects of vB_CbrM_HP1 *in vivo* and *in vitro*. Compared with the above five phages, vB_CbrM_HP1 had obvious pH and temperature stability. The *C. freundii* phages LK1 and CfP1 became completely inactive at a pH of less than 4 (20). Phage LK1 became completely inactive at 70°C (20). Phage CfP1 became completely inactive at 60°C (22). Studies have shown that phages without tail show a wider host range than those with tail (55). The host range is probably related to receptor-binding proteins (RBPs), such as extended tail fibers, tail spikes and the central tail spike proteins (56, 57). The difference in phage infection ability of different strains of the same species is related to the type of defense mechanism they employ (58).

In this study, the tail proteins of vB_CbrM_HP1 were probably encoded by the ORFs ORF16, ORF52, ORF75, and ORF97, which had very low similarity by sequence alignment on NCBI. We found that the halo around plaques may be due to the presence of polysaccharide depolymerizing enzymes (PDS) in phages. PDS are proteins that can be attached to the tail of phages. They can degrade capsular polysaccharides (CPS), exopolysaccharides (EPS) and lipopolysaccharides (LPS) (59). Through gene analysis, we found that these enzymes encoded by the ORFs ORF16 ORF52, ORF75, and ORF97 might be involved in the production of PDS (60). Double-stranded DNA phages that encode at least one holin generally use the holin-endolysin system to lyse bacteria (61). To ensure that phages have sufficient resources to synthesize progeny phages, the synthesis of endolysin must be controllable (62). By comparison with the sequences in the NCBI database, ORF14 (99% identity; 99% positives) and ORF110 (89% identity; 95% positives) were found to possibly encode endolysin proteins. The size of these two proteins was approximately 20 kDa. A large amount of endolysin accumulated in the cell before lysis of the host and did not cross the cell membrane to reach the target cell wall. Thus, endolysin does not have the characteristics of an exocrine (63). Holin, a small membrane protein, was the second lysis factor that degraded the cell membrane. It could control the timing of lysis (61). The phage holin protein was probably encoded by ORF24 (86% identity; 92% positives) or ORF54 (99% identity; 100% positives), which had high similarity by sequence alignment on NCBI. The protein size encoded by ORF14 was approximately 22 kDa and that encoded by ORF54 was approximately 13 kDa. Different from *Staphylococcus aureus* phage GH15 (**JQ686190)** (64) and *Citrobacter freundii* phage IME-JL8 (**MT02308**) (19), the holin of phage vB_CbrM_HP1 was not adjacent to endolysin. The endolysin of vB_CbrM_HP1 was adjacent to the tail protein. Similar phenomena were also found in *Shigella* phage SF17 (**KM236239**) (46) and *Citrobacter* phage Mijalis (**KY654690**) (52), which were more than 90% similar to vB_CbrM_HP1. Through sequence alignment, we preliminarily speculated the function of the proteins encoded by ORF14, ORF110, ORF24 and ORF54. Whether they are endolysin or holin proteins remains to be verified by further experiments. As components of the lysis system, the endolysin and holin proteins inferred in this study have the potential to be used as a delivery system to infuse drugs, nucleic acids, and proteins into eukaryotic cells (65).

vB_CbrM_HP1 had a good antibacterial effect *in vitro* and *in vivo*. The antibacterial effect *in vitro* was consistent with that of *Yersinia* phage X1 (31). Several reports have described successful phage therapies. Treatment with phage pAh6.2TG has been shown to significantly increase the survival (50–75%) of Nile tilapia exposed to a lethal dose of pathogenic *A. hydrophila* MDR (66). Furthermore, treatment with 1 × 10^8^ PFU/ml phage AKH-2 improved the survival rate of Nile tilapia (41.1%) (67). To assess the therapeutic effect of vB_CbrM_HP1, different phage doses were administered intraperitoneally 1 h after bacterial infection. All the carps administrated with phage vB_CbrM_HP1 (1 × 10^9^ PFU/fish) recovered. Our study showed that the survival rate of crucian carp infected with the lethal bacteria could even be improved by the phage treatment at 12 h and 24 h after the challenge.

Based on histopathological analysis, the normal tissues of the gut, liver and spleen showed intact structures and no inflammatory cell infiltration. However, our study showed that intraperitoneal injection of *C. braakii* had serious effects on the gut and spleen of crucian carp. Compared with the control group of crucian carp, the inflammation and pathological changes were significantly alleviated in the gut and spleen tissues from crucian carp that were treated with phage. Furthermore, compared with the control group, vB_CbrM_HP1 administration resulted in a decrease in cytokine (TNF-α, IFN-γ, IL-1β) levels which was the same phenomenon as seen in the phage PIZ SAE-01E2 and the phage AVP in the treatment of infection with *S. abortus equi and A. viridans*, respectively (33, 68). These results showed that phage vB_CbrM_HP1 can effectively alleviate lesions *in vivo*. Some studies have shown that phages have anti-inflammatory properties that reduce the secretion of pro-inflammatory factors (69, 70). We are trying to isolate several new *Citrobacter* phages to develop phage cocktails or combination treatments of *Citrobacter* phages with antibiotics to broaden the host range of these treatments and better prevent and treat *C. braakii* infection.

## Conclusions

In summary, we isolated the *C. braakii* phage vB_CbrM_HP1 for the first time. *In vitro*, it had relatively steady temperature and pH characteristics and obvious antibacterial effects. These promising characteristics provided a driving force for further exploring its antibacterial effect *in vivo*. *In vivo*, administration of vB_CbrM_HP1 1 h after infection achieved 100% protection in the crucian carp model. Also, vB_CbrM_HP1 treatment effectively alleviated pathological damage in the gut and spleen and the levels of inflammatory factors in the gut. The results of our study provide a new treatment idea for *C. braakii* infection, and follow-up experiments should be performed.

## Data Availability Statement

The datasets presented in this study can be found in online repositories. The names of the repository/repositories and accession number(s) can be found in the article/Supplementary Material.

## Ethics Statement

The animal study was reviewed and approved by the Animal Welfare and Research Ethics Committee at Jilin Agriculture University.

## Author Contributions

LZ and ZG conceived and designed the experiments. CH, CF, XL, and RZ performed the experiments. JG and WH wrote the manuscript. CH, ZW, and HX conducted data collection and analysis and drafted the manuscript. HO read and revised the manuscript. All authors contributed to the article and approved the submitted version.

## Funding

This work was financially supported through grants from the National Natural Science Foundation of China (No. 81802056), China Postdoctoral Science Foundation (No. 2019M651215), the Achievement Transformation Project of the First Hospital of Jilin University (No. JDYYGH2019013), and the Jilin Province Science and technology development plan project (No. 20210508006RQ).

## Conflict of Interest

The authors declare that the research was conducted in the absence of any commercial or financial relationships that could be construed as a potential conflict of interest.

## Publisher's Note

All claims expressed in this article are solely those of the authors and do not necessarily represent those of their affiliated organizations, or those of the publisher, the editors and the reviewers. Any product that may be evaluated in this article, or claim that may be made by its manufacturer, is not guaranteed or endorsed by the publisher.
