# Supplementary material for: The Bacteriophage vB_CbrM_HP1 Protects Crucian Carp Against Citrobacter braakii Infection
Source: Front Vet Sci. 2022 May 6;9:888561. doi: 10.3389/fvets.2022.888561 (PMC9120918; doi:10.3389/fvets.2022.888561)
Supplement: Supplementary file 1 [file Table_1.DOCX]

Table S1. The host range of vB_CbrM_HP1.

| **Strain No**. | **Bacterial strain** | **Spot of vB_CbrM_HP1** |
| --- | --- | --- |
| 1 | *Escherichia coli* AE-03-1^1^ | N |
| 2 | *Escherichia coli* AE-16-1^1^ | N |
| 3 | *Escherichia coli* AE-17-1^1^ | N |
| 4 | *Escherichia coli* AE-23-2^1^ | N |
| 5 | *Escherichia coli* AE-31-1^1^ | N |
| 6 | *Escherichia coli* BE-13-1^1^ | N |
| 7 | *Escherichia coli* CE-36-2^1^ | N |
| 8 | *Escherichia coli* EE-44^1^ | N |
| 9 | *Salmonella* GS-27^1^ | N |
| 10 | *Salmonella* HS-39^1^ | N |
| 11 | *Salmonella* KS-17-1^1^ | N |
| 12 | *Salmonella* MS-32^1^ | N |
| 13 | *Salmonella* AE-30-2^1^ | N |
| 14 | *Salmonella* WE-23-1^1^ | N |
| 15 | *Salmonella* AS-3-2^1^ | N |
| 16 | *Salmonella* ES-44^1^ | N |
| 17 | *Citrobacter freundii* IE-10^1^ | Y |
| 18 | *Citrobacter freundii* IE-13^1^ | N |
| 19 | *Citrobacter freundii* KE-13-2^1^ | N |
| 20 | *Citrobacter freundii* ZE-68^1^ | N |
| 21 | *Citrobacter freundii* ZE-70^1^ | N |
| 22 | *Citrobacter freundii* ZE-98^1^ | N |
| 23 | *Citrobacter freundii* LGE-2^1^ | N |
| 24 | *Citrobacter freundii* AS-47-2^1^ | N |
| 25 | *Citrobacter freundii* AE-46-2^1^ | N |
| 26 | *Citrobacter freundii* BE-28-2^1^ | N |
| 27 | *Citrobacter freundii* BE-29-2^1^ | N |
| 28 | *Citrobacter freundii* ZE-26^1^ | N |
| 29 | *Citrobacter freundii* ZE-94^1^ | N |
| 30 | *Citrobacter freundii* AS-1-3^1^ | Y |
| 31  32 | *Citrobacter freundii* JS-17^1^  *Citrobacter freundii* OS-11^1^ | N  N |
| 33 | *Citrobacter freundii* 5^2^ | N |
| 34 | *Citrobacter freundii* 15^2^ | Y |
| 35 | *Citrobacter freundii* 77^2^ | N |
| 36 | *Citrobacter freundii* 78^2^ | N |
| 37 | *Citrobacter freundii* 79^2^ | N |
| 38 | *Citrobacter freundii* 223^2^ | N |
| 39 | *Citrobacter freundii* 1136^2^ | N |
| 40 | *Citrobacter freundii* 1152^2^ | N |
| 41 | *Citrobacter freundii* 1864^2^ | N |
| 42 | *Citrobacter freundii* 2052^2^ | N |
| 43 | *Citrobacter freundii* 2151^2^ | N |
| 44 | *Citrobacter freundii* 2262^2^ | N |
| 45 | *Citrobacter braakii* Cbr-1^2^ | Y |
| 46 | *Citrobacter braakii* JS-17^3^ | N |
| 47 | *Citrobacter braakii* XS-31^3^ | N |
| 48 | *Citrobacter braakii* AE-47-1^3^ | N |
| 49 | *Citrobacter braakii* BE-32-2^3^ | N |
| 50 | *Citrobacter braakii* XS-31^3^ | N |

Y: lysis N: no lysis

^1^ Isolated from the swine and stored in Jingmin Gu’s lab.

^2^ Isolated from the carp and stored in Lei Zhang’s lab.

^3^ Given by professor Xiaofeng Shan.
