# Supplementary material for: The Bacteriophage vB_CbrM_HP1 Protects Crucian Carp Against Citrobacter braakii Infection
Source: Front Vet Sci. 2022 May 6;9:888561. doi: 10.3389/fvets.2022.888561 (PMC9120918; doi:10.3389/fvets.2022.888561)
Supplement: Supplementary file 2 [file Table_2.DOCX]

Table S2. Sequences and conditions of the primers used in qPCR analysis.

| **Gene** | **Nucleotide Sequence (5'-3')** | **Annealing Temp (°C)** | **NCBI Accession No.** |
| --- | --- | --- | --- |
| IL-1β | F: AACTGATGACCCGAATGGAAAC | 55 | AY340959.1 |
|  | R: CACCTTCTCCCAGTCGTCAAA |  |  |
| TNF-α | F: TTATGTCGGTGCGGCCTTC | 55 | AY427649.1 |
|  | R: AGGTCTTTCCGTTGTCGCTTT |  |  |
| IFN-γ | F: AACAGTCGGGTGTCGCAAG | 60 | EU909368.1 |
|  | R: TCAGCAAACATACTCCCCA |  |  |
| β-actin | F: CAAGATGATGGTGTGCCAAGTG | 58 | AF025305 |
|  | R: TCTGTCTCCGGCACGAAGTA |  |  |
