# Supplementary material for: The Bacteriophage vB_CbrM_HP1 Protects Crucian Carp Against Citrobacter braakii Infection
Source: Front Vet Sci. 2022 May 6;9:888561. doi: 10.3389/fvets.2022.888561 (PMC9120918; doi:10.3389/fvets.2022.888561)
Supplement: Supplementary file 3 [file Table_3.DOCX]

Table S3. The ORFs analysis of vB_CbrM_HP1 genome.

| ORF | Start-End | | Length  (aa) | Protein size (kDa) | pI | Annotated function | Representative similarity to proteins in database | E  value | Query  cover | Identity  (positives) | Accession no. |  |
| --- | --- | --- | --- | --- | --- | --- | --- | --- | --- | --- | --- | --- |
| 1^a^- | 1209 | 1868 | 219 | 25.663 | 7.78 | hypothetical protein | Shigella flexneri | 5e-156 | 100% | 97%(98%) | EFV7170034.1 |  |
| 2^a^- | 1880 | 2338 | 152 | 17.463 | 5.15 | hypothetical protein | Shigella flexneri | 1e-103 | 99% | 96%(98%) | EFV7170035.1 |  |
| 3^a^- | 2322 | 2618 | 98 | 11.305 | 4.25 | hypothetical protein FDI02_gp035 | Citrobacter phage Mordin | 2e-66 | 100% | 98%(98%) | YP_009606565.1 |  |
| 4^a^- | 2660 | 2869 | 69 | 7.933 | 6.70 | hypothetical protein | Citrobacter freundii | 2e-40 | 100% | 100%(100%) | HAU5726687.1 |  |
| 5^a^- | 2866 | 2958 | 30 | 3.460 | 8.93 | hypothetical protein CPT_Michonne_gp124 | Citrobacter phage Michonne | 6e-26 | 100% | 100%(100%) | YP_009177352.1 |  |
| 6^a^- | 2940 | 3446 | 168 | 19.666 | 6.61 | hypothetical protein | Citrobacter freundii | 3e-117 | 100% | 97%(98%) | HAU5726688.1 |  |
| 7^a^- | 3443 | 3661 | 72 | 8.597 | 10.22 | hypothetical protein CPT_Moogle98 | Citrobacter phage Moogle | 6e-45 | 100% | 99%(100%) | YP_009145741.1 |  |
| 8^a^- | 3658 | 4383 | 241 | 27.675 | 5.36 | hypothetical protein | Citrobacter freundii | 3e-155 | 100% | 88%(93%) | HAU5726690.1 |  |
| 9^a^- | 4454 | 5044 | 196 | 22.461 | 5.62 | hypothetical protein | Citrobacter freundii | 9e-144 | 100% | 100%(100%) | HAU5726691.1 |  |
| 10^a^- | 5046 | 5390 | 114 | 13.241 | 9.50 | hypothetical protein CPT_Moogle95 | Citrobacter phage Moogle | 1e-75 | 100% | 99%(100%) | YP_009145738.1 |  |
| 11^a^- | 5383 | 5676 | 97 | 11.296 | 4.53 | hypothetical protein CPT_Moogle94 | Citrobacter phage Moogle | 4e-64 | 100% | 100%(100%) | YP_009145737.1 |  |
| 12^a^- | 5678 | 6061 | 127 | 14.730 | 4.37 | hypothetical protein | Escherichia coli | 2e-87 | 100% | 98%(99%) | EFG4626582.1 |  |
| 13^a^- | 6054 | 6452 | 132 | 15.418 | 6.62 | hypothetical protein | Escherichia coli | 9e-93 | 100% | 100%(100%) | EFG4626583.1 |  |
| 14^a^- | 6504 | 6968 | 154 | 17.008 | 9.28 | Lysozyme | Citrobacter phage | 4e-109 | 100% | 99%(99%) | [YP_009606577.1](https://www.ncbi.nlm.nih.gov/protein/YP_009606577.1?report=genbank&log$=protalign&blast_rank=1&RID=RWK9E6DJ013) |  |
| 15^a^- | 6970 | 7854 | 294 | 31.400 | 4.63 | tail protein | Shigella phage Sf13 | 0.0 | 99% | 92%(95%) | YP_009612635.1 |  |
| 16^a^- | 7851 | 8219 | 122 | 14.204 | 6.36 | tail protein | Escherichia phage vB_EcoM-Pr103Blw | 1e-64 | 100% | 75%(88%) | QSL98980.1 |  |
| 17^a^- | 8263 | 8853 | 196 | 22.135 | 4.91 | hypothetical protein CPT_Moogle88 | Citrobacter phage Moogle88 | 2e-135 | 100% | 96%(98%) | YP_009145731.1 |  |
| 18^a^- | 8847 | 9080 | 77 | 8.780 | 8.31 | hypothetical protein CPT_Moogle87 | Citrobacter phage Moogle | 5e-49 | 100% | 100%(100%) | [YP_009145730.1](https://www.ncbi.nlm.nih.gov/protein/YP_009145730.1?report=genbank&log$=protalign&blast_rank=1&RID=RWVUCWVS013) |  |
| 19^a^- | 9602 | 9223 | 53 | 6.426 | 9.41 | Hypothetical protein CPT_Michonne_gp138 | Citrobacter phage Michonne | 5e-29 | 100% | 96%(96%) | [YP_009177338.1](https://www.ncbi.nlm.nih.gov/protein/YP_009177338.1?report=genbank&log$=protalign&blast_rank=1&RID=RWW02TZT013) |  |
| 20^a^+ | 9473 | 9844 | 123 | 13.129 | 9.96 | hypothetical protein CPT_Moogle86 | Citrobacter phage Moogle | 4e-83 | 100% | 100%(100%) | [YP_009145729.1](https://www.ncbi.nlm.nih.gov/protein/YP_009145729.1?report=genbank&log$=protalign&blast_rank=1&RID=RWW8AXEH013) |  |
| 21^a^+ | 9921 | 11336 | 471 | 53.241 | 8.22 | DUF2828 domain-containing protein | Shigella flexneri | 0.0 | 100% | 95%(98%) | [EGE3641522.1](https://www.ncbi.nlm.nih.gov/protein/EGE3641522.1?report=genbank&log$=protalign&blast_rank=13&RID=RWWF0SGX013) |  |
| 22^c^+ | 12278 | 12406 | 42 | 4.904 | 10.17 | No |  |  |  |  |  |  |
| 23^a^+ | 14835 | 15062 | 75 | 8.851 | 7.98 | hypothetical protein FDJ00_gp137 | Shigella phage Sf17 | 1e-46 | 100% | 95%(97%) | [YP_009618701.1](https://www.ncbi.nlm.nih.gov/protein/YP_009618701.1?report=genbank&log$=protalign&blast_rank=1&RID=RWWWX5CR013) |  |
| 24^a^+ | 15059 | 15619 | 186 | 21.929 | 10.00 | Holin | Salmonella phage vB_Si_35FD | 9e-116 | 98% | 86%(92%) | [QZD26249.1](https://www.ncbi.nlm.nih.gov/protein/QZD26249.1?report=genbank&log$=protalign&blast_rank=12&RID=RWX2R1CY013) |  |
| 25^c^+ | 16075 | 16809 | 244 | 25.339 | 4.13 | putative membrane protein | Shigella phage KPS64 | 5e-156 | 100% | 95%(98%) | [QBP32848.1](https://www.ncbi.nlm.nih.gov/protein/QBP32848.1?report=genbank&log$=protalign&blast_rank=9&RID=RWXANG5H016) |  |
| 26^a^+ | 17204 | 17410 | 68 | 7.762 | 4.19 | hypothetical protein CPT_Moogle82 | Citrobacter phage Moogle | 3e-40 | 100% | 100%(100%) | YP_009145725.1 |  |
| 27^a^+ | 17432 | 19033 | 533 | 60.054 | 6.94 | terminase large subunit | Citrobacter phage Moogle | 0.0 | 100% | 100%(100%) | [YP_009145724.1](https://www.ncbi.nlm.nih.gov/protein/YP_009145724.1?report=genbank&log$=protalign&blast_rank=1&RID=RWXXXE53016) |  |
| 28^a^+ | 19046 | 20512 | 488 | 55.323 | 6.30 | putative portal protein | Shigella phage Sf14 | 0.0 | 100% | 96%(98%) | [YP_009618574.1](https://www.ncbi.nlm.nih.gov/protein/YP_009618574.1?report=genbank&log$=protalign&blast_rank=9&RID=RWYA6DSH016) |  |
| 29^b^+ | 20512 | 21012 | 166 | 18.385 | 6.36 | hypothetical protein CPT_Michonne_gp082 | Citrobacter phage Michonne | 3e-117 | 100% | 99%(100%) | [YP_009177329.1](https://www.ncbi.nlm.nih.gov/protein/YP_009177329.1?report=genbank&log$=protalign&blast_rank=1&RID=RZ0JTGSE013) |  |
| 30^a^+ | 21012 | 21344 | 110 | 11.628 | 4.49 | hypothetical protein CPT_Moogle78 | Citrobacter phage Moogle | 4e-73 | 100% | 100%(100%) | [YP_009145721.1](https://www.ncbi.nlm.nih.gov/protein/YP_009145721.1?report=genbank&log$=protalign&blast_rank=1&RID=RZ0T64MX016) |  |
| 31^a^+ | 21356 | 22693 | 445 | 47.965 | 4.59 | signal peptide peptidase SppA | Citrobacter freundii | 0.0 | 100% | 96%(97%) | HAU5726711.1 |  |
| 32^a^+ | 22706 | 23083 | 125 | 13.617 | 5.03 | major capsid protein | Shigella phage vB_SflM_004 | 3e-43 | 63% | 87%(91%) | [AZV01486.1](https://www.ncbi.nlm.nih.gov/protein/AZV01486.1?report=genbank&log$=protalign&blast_rank=85&RID=RZ17V6XV016) |  |
| 33^a^+ | 23115 | 24221 | 368 | 41.675 | 5.38 | major capsid protein | Citrobacter phage Michonne | 0.0 | 99% | 98%(99%) | [YP_009177325.1](https://www.ncbi.nlm.nih.gov/protein/YP_009177325.1?report=genbank&log$=protalign&blast_rank=2&RID=RZ1CBG67016) |  |
| 34^a^+ | 24244 | 24693 | 149 | 17.015 | 6.53 | hypothetical protein CPT_Moogle74 | Citrobacter phage Moogle | 4e-105 | 100% | 100%(100%) | [YP_009145717.1](https://www.ncbi.nlm.nih.gov/protein/YP_009145717.1?report=genbank&log$=protalign&blast_rank=1&RID=RZM2C6AM013) |  |
| 35^a^+ | 24693 | 25175 | 160 | 17.76 | 10.14 | tail fibers protein | Escherichia phage vB_EcoM-Pr103Blw | 3e-107 | 100% | 94%(98%) | [QSL98997.1](https://www.ncbi.nlm.nih.gov/protein/QSL98997.1?report=genbank&log$=protalign&blast_rank=25&RID=RZM5TFXT016) |  |
| 36^a^+ | 25172 | 25573 | 133 | 15.482 | 5.62 | hypothetical protein CPT_Moogle72 | Citrobacter phage Moogle | 4e-94 | 100% | 100%(100%) | [YP_009145715.1](https://www.ncbi.nlm.nih.gov/protein/YP_009145715.1?report=genbank&log$=protalign&blast_rank=1&RID=RZMC4M4V013) |  |
| 37^a^+ | 25548 | 26174 | 199 | 22.282 | 4.61 | hypothetical protein CPT_Moogle71 | Citrobacter phage Moogle | 4e-144 | 100% | 100%(100%) | [YP_009145714.1](https://www.ncbi.nlm.nih.gov/protein/YP_009145714.1?report=genbank&log$=protalign&blast_rank=1&RID=RZMFSV63013) |  |
| 38^a^+ | 26148 | 27500 | 450 | 48.784 | 4.97 | DUF3383 family protein | Citrobacter phage Moogle | 0.0 | 100% | 98%(99%) | [YP_009145713.1](https://www.ncbi.nlm.nih.gov/protein/YP_009145713.1?report=genbank&log$=protalign&blast_rank=1&RID=RZMHRD50016) |  |
| 39^c^+ | 27516 | 27962 | 148 | 16.195 | 4.11 | DUF3277 domain-containing protein | Verrucomicrobia bacterium | 3e-09 | 51% | 31%(47%) | [PWU04930.1](https://www.ncbi.nlm.nih.gov/protein/PWU04930.1?report=genbank&log$=protalign&blast_rank=79&RID=RZMNFF3D013) |  |
| 40^a^+ | 28039 | 28440 | 133 | 14.698 | 4.65 | tape measure chaperone | Citrobacter phage Moogle | 2e-89 | 100% | 99%(99%) | [YP_009145711.1](https://www.ncbi.nlm.nih.gov/protein/YP_009145711.1?report=genbank&log$=protalign&blast_rank=2&RID=RZMNYCX5013) |  |
| 41^a^+ | 28449 | 28676 | 75 | 8.679 | 4.41 | tape measure  chaperone | Citrobacter phage Moogle | 6e-46 | 100% | 100%(100%) | YP_009145710.1 |  |
| 42^a^+ | 28676 | 30904 | 742 | 80.278 | 9.65 | tail length | Citrobacter phage Michonne | 0.0 | 100% | 98%(99%) | [YP_009177316.1](https://www.ncbi.nlm.nih.gov/protein/YP_009177316.1?report=genbank&log$=protalign&blast_rank=1&RID=RZN16913013) |  |
| 43^a^+ | 30904 | 31710 | 268 | 28.909 | 7.81 | hypothetical protein CPT_Moogle65 | Citrobacter phage Moogle | 0.0 | 100% | 99%(99%) | [YP_009145708.1](https://www.ncbi.nlm.nih.gov/protein/YP_009145708.1?report=genbank&log$=protalign&blast_rank=1&RID=RZN1UJCK013) |  |
| 44^a^+ | 31710 | 32051 | 113 | 12.920 | 4.75 | hypothetical protein CPT_Moogle64 | Citrobacter phage Moogle | 6e-78 | 100% | 100%(100%) | YP_009145707.1 |  |
| 45^a^+ | 32051 | 33028 | 325 | 37.133 | 8.83 | hypothetical protein | Citrobacter phage Michonne | 0.0 | 100% | 100%(100%) | YP_009177313.1 |  |
| 46^a^+ | 33025 | 33666 | 213 | 23.651 | 4.58 | baseplate protein | Citrobacter freundii | 5e-155 | 100% | 97%(98%) | [HAU5726726.1](https://www.ncbi.nlm.nih.gov/protein/HAU5726726.1?report=genbank&log$=protalign&blast_rank=3&RID=RZNA2ZT4013) |  |
| 47^a^+ | 33666 | 34082 | 138 | 15.509 | 7.23 | hypothetical protein CPT_Moogle61 | Citrobacter phage Moogle | 6e-97 | 100% | 100%(100%) | [YP_009145704.1](https://www.ncbi.nlm.nih.gov/protein/YP_009145704.1?report=genbank&log$=protalign&blast_rank=1&RID=RZNCMJV8013) |  |
| 48^a^+ | 34082 | 35551 | 489 | 53.335 | 4.29 | baseplate assembly protein | Citrobacter phage Moogle | 0.0 | 100% | 99%(99%) | [YP_009145703.1](https://www.ncbi.nlm.nih.gov/protein/YP_009145703.1?report=genbank&log$=protalign&blast_rank=1&RID=RZND4V1S013) |  |
| 49^a^+ | 35554 | 36411 | 285 | 31.451 | 5.16 | putative baseplate assembly protein | Escherichia phage mistaenkt | 2e-180 | 100% | 85%(93%) | [QHR67974.1](https://www.ncbi.nlm.nih.gov/protein/QHR67974.1?report=genbank&log$=protalign&blast_rank=13&RID=RZNDYY3U016) |  |
| 50^a^+ | 36411 | 36710 | 99 | 10.997 | 4.23 | hypothetical protein | Citrobacter freundii | 2e-65 | 100% | 99%(98%) | HAU5726730.1 |  |
| 51^a^+ | 36713 | 37921 | 402 | 42.737 | 6.63 | phage tail protein | Citrobacter freundii | 0.0 | 100% | 99%(100%) | HAU5726731.1 |  |
| 52^a^+ | 37967 | 40492 | 841 | 89.986 | 6.91 | phage tail protein | Citrobacter freundii | 0.0 | 100% | 76%(85%) | [HAU5726732.1](https://www.ncbi.nlm.nih.gov/protein/HAU5726732.1?report=genbank&log$=protalign&blast_rank=1&RID=RZNWKS36016) |  |
| 53^b^+ | 40579 | 40773 | 64 | 7.495 | 6.58 | hypothetical protein CPT_Moogle55 | Citrobacter phage Moogle | 2e-37 | 100% | 100%(100%) | [YP_009145698.1](https://www.ncbi.nlm.nih.gov/protein/YP_009145698.1?report=genbank&log$=protalign&blast_rank=1&RID=RZNY0SX2013) |  |
| 54^a^+ | 40773 | 41147 | 124 | 13.805 | 8.89 | putative holin | Citrobacter phage | 2e-82 | 100% | 99%(100%) | YP_009145697.1 |  |
| 55^a^- | 41189 | 42088 | 299 | 34.235 | 6.04 | thymidylate synthase  dTMP synthase | Citrobacter freundii | 0.0 | 100% | 99%(100%) | HAU5726735.1 |  |
| 56^a^- | 42090 | 42635 | 181 | 20.360 | 4.98 | Dihydrofolate reductase | Citrobacter freundii | 1e-129 | 100% | 99%(100%) | HAU5726736.1 |  |
| 57^a^- | 42094 | 42632 | 90 | 10.145 | 6.71 | hypothetical protein CPT_Moogle51 | Citrobacter phage Moogle | 5e-60 | 100% | 98%(100%) | YP_009145694.1 |  |
| 58^a^- | 42901 | 43416 | 171 | 19.458 | 4.55 | hypothetical protein CPT_Michonne_gp053protein | Citrobacter phage Michonne | 2e-120 | 100% | 98%(100%) | [YP_009177300.1](https://www.ncbi.nlm.nih.gov/protein/YP_009177300.1?report=genbank&log$=protalign&blast_rank=1&RID=RZPAX1SN013) |  |
| 59^a^- | 43418 | 43921 | 167 | 19.713 | 7.94 | hypothetical protein | Shigella flexneri | 9e-121 | 100% | 99%(100%) | EFV7169969.1 |  |
| 60^a^- | 43906 | 44265 | 119 | 13.493 | 7.33 | hypothetical protein | Citrobacter phage Michonne | 3e-80 | 100% | 97%(98%) | YP_009177298.1 |  |
| 61^a^- | 44267 | 44566 | 99 | 11.446 | 4.29 | XRE family transcriptional regulator | Citrobacter freundii | 5e-66 | 100% | 100%(100%) | HAU5726741.1 |  |
| 62^a^- | 44559 | 44762 | 67 | 7.798 | 5.15 | hypothetical protein | Citrobacter freundii | 1e-39 | 100% | 100%(100%) | HAU5726742.1 |  |
| 63^a^- | 44765 | 45145 | 126 | 14.103 | 4.86 | hypothetical protein CPT_Moogle45 | Citrobacter phage Moogle | 6e-86 | 100% | 97%(99%) | YP_009145688.1 |  |
| 64^a^- | 45387 | 46487 | 366 | 42.467 | 7.14 | DNA ligase | Shigella phage Sf14 | 0.0 | 100% | 97%(98%) | YP_009618610.1 |  |
| 65^a^- | 46569 | 46811 | 80 | 9.435 | 4.11 | hypothetical protein | Escherichia coli | 3e-47 | 100% | 90%(95%) | [EFG4626632.1](https://www.ncbi.nlm.nih.gov/protein/EFG4626632.1?report=genbank&log$=protalign&blast_rank=1&RID=S1WSBWDZ013) |  |
| 66^a^- | 46808 | 47104 | 68 | 8.171 | 7.30 | hypothetical protein CPT_Moogle41 | Citrobacter phage Moogle | 1e-40 | 100% | 97%(100%) | YP_009145684.1 |  |
| 67^a^- | 47025 | 47261 | 78 | 8.825 | 8.30 | hypothetical protein CPT_Moogle40 | Citrobacter phage Moogle | 4e-51 | 100% | 99%(98%) | YP_009145683.1 |  |
| 68^a^- | 47469 | 47612 | 47 | 5.856 | 9.05 | hypothetical protein KPS64_gp40 | Shigella phage KPS64 | 2e-25 | 100% | 100%(100%) | QBP32803.1 |  |
| 69^a^- | 47599 | 47790 | 63 | 7.437 | 4.75 | hypothetical protein KPS64_gp39 | Shigella phage KPS64 | 7e-36 | 100% | 90%(96%) | QBP32802.1 |  |
| 70^a^- | 47787 | 47960 | 57 | 6.562 | 9.50 | hypothetical protein dune_65 | Escherichia phage dune | 4e-06 | 94% | 44%(61%) | QHR74346.1 |  |
| 71^a^- | 47957 | 48166 | 69 | 8.109 | 10.44 | hypothetical protein CPT_Moogle38 | Citrobacter phage Moogle | 5e-43 | 100% | 100%(100%) | [YP_009145681.1](https://www.ncbi.nlm.nih.gov/protein/YP_009145681.1?report=genbank&log$=protalign&blast_rank=1&RID=S1X67C1W016) |  |
| 72^a^- | 48163 | 48381 | 72 | 8.117 | 5.82 | hypothetical protein CPT_Michonne_gp0  38 | Citrobacter phage Michonne | 8e-43 | 100% | 97%(100%) | YP_009177285.1 |  |
| 73^a^- | 48442 | 51141 | 899 | 103.259 | 7.96 | DNA polymerase | Citrobacter phage Moogle | 0.0 | 100% | 99%(99%) | YP_009606632.1 |  |
| 74^a^+ | 51401 | 51841 | 146 | 17.457 | 5.47 | hypothetical protein CPT_Moogle35 | Citrobacter phage Moogle | 9e-103 | 100% | 99%(99%) | YP_009145678.1 |  |
| 75^a^+ | 51825 | 52619 | 264 | 29.165 | 4.45 | minor tail protein | Shigella phage Z31 | 3e-61 | 100% | 41%(67%) | YP_009145677.1 |  |
| 76^a^+ | 52676 | 53416 | 246 | 28.026 | 8.35 | nucleoside triphosphate hydrolase | Citrobacter phage Moogle | 2e-178 | 100% | 99%(99%) | YP_009145676.1 |  |
| 77^a^+ | 53429 | 53620 | 63 | 7.419 | 7.33 | hypothetical protein | Escherichia coli | 3e-35 | 100% | 100%(100%) | EFG4626517.1 |  |
| 78^a^+ | 53613 | 55604 | 663 | 74.902 | 5.30 | DNA helicase | Citrobacter phage Moogle | 0.0 | 100% | 98%(99%) | [ATE85982.1](https://www.ncbi.nlm.nih.gov/protein/ATE85982.1?report=genbank&log$=protalign&blast_rank=3&RID=S1Y8FM4F013) |  |
| 79^a^+ | 55579 | 55857 | 92 | 11.446 | 7.33 | hypothetical protein  regulator | Citrobacter freundii | 3e-60 | 100% | 99%(100%) | HAU5726754.1 |  |
| 80^a^+ | 55854 | 55991 | 45 | 4.952 | 10.64 | hypothetical protein CPT_Moogle29 | Citrobacter phage Moogle | 1e-21 | 100% | 100%(100%) | YP_009145672.1 |  |
| 81^a^+ | 56078 | 56941 | 287 | 32.059 | 4.33 | hypothetical protein CPT_Michonne_gp028 | Citrobacter phage Michonne | 0.0 | 100% | 99%(99%) | [YP_009177275.1](https://www.ncbi.nlm.nih.gov/protein/YP_009177275.1?report=genbank&log$=prottop&blast_rank=1&RID=S1Y9XRNZ016) |  |
| 82^a^+ | 57005 | 58045 | 346 | 39.736 | 5.66 | Exodeoxyribonucleae | Citrobacter freundii | 0.0 | 100% | 100%(100%) | [HAU5726756.1](https://www.ncbi.nlm.nih.gov/protein/HAU5726756.1?report=genbank&log$=prottop&blast_rank=1&RID=S1YACBGH013) |  |
| 83^a^+ | 58035 | 58535 | 166 | 19.599 | 9.50 | GIY-YIG homing endonuclease | Citrobacter phage Moogle | 3e-117 | 100% | 99%(99%) | [YP_009145668.1](https://www.ncbi.nlm.nih.gov/protein/YP_009145668.1?report=genbank&log$=prottop&blast_rank=1&RID=S1YAS839013) |  |
| 84^a^+ | 58537 | 58752 | 71 | 8.169 | 6.37 | hypothetical protein CPT_Michonne_gp025 | Citrobacter phage Michonne | 2e-41 | 100% | 99%(100%) | [YP_009177272.1](https://www.ncbi.nlm.nih.gov/protein/YP_009177272.1?report=genbank&log$=prottop&blast_rank=1&RID=S1YB54K9016) |  |
| 85^a^+ | 58752 | 59057 | 251 | 28.759 | 4.72 | putative phosphoribosyl-ATP pyrophosphohydrolase-like protein | Shigella phage Sf18 | 2e-173 | 100% | 92%(96%) | [ATE86312.1](https://www.ncbi.nlm.nih.gov/protein/ATE86312.1?report=genbank&log$=prottop&blast_rank=9&RID=S202JBG9013) |  |
| 86^a^+ | 59488 | 59814 | 108 | 12.558 | 5.25 | Putative deoxyribonucleoside reductase | [Shigella phage Sf18](http://www.uniprot.org/taxonomy/2024319) | 2e-64 | 100% | 92.6 (100) | ATE86313.1 |  |
| 87^a^+ | 59807 | 60142 | 111 | 12.887 | 8.72 | loader of DNA helicase | Escherichia phage SP22 | 3e-26 | 100% | 45%(65%) | [BBU53715.1](https://www.ncbi.nlm.nih.gov/protein/BBU53715.1?report=genbank&log$=prottop&blast_rank=71&RID=S203C4Z1016) |  |
| 88^a^+ | 60193 | 62427 | 744 | 85.212 | 7.78 | ribonucleoside triphosphate reductase large subunit | Citrobacter phage Michonne | 0.0 | 100% | 99%(100%) | [YP_009177268.1](https://www.ncbi.nlm.nih.gov/protein/YP_009177268.1?report=genbank&log$=prottop&blast_rank=1&RID=S203TBUD013) |  |
| 89^a^+ | 62465 | 62740 | 91 | 10.977 | 5.70 | hypothetical protein | Escherichia coli | 6e-62 | 100% | 100%(100%) | [EFG4626528.1](https://www.ncbi.nlm.nih.gov/protein/EFG4626528.1?report=genbank&log$=prottop&blast_rank=1&RID=S2048AB6016) |  |
| 90^a^+ | 62737 | 63810 | 357 | 41.291 | 4.65 | ribonucleoside triphosphate reductase small subunit | Citrobacter phage Michonne | 0.0 | 100% | 99%(99%) | [YP_009177266.1](https://www.ncbi.nlm.nih.gov/protein/YP_009177266.1?report=genbank&log$=prottop&blast_rank=1&RID=S204S05H016) |  |
| 91^a^+ | 63810 | 64052 | 80 | 9.070 | 5.76 | putative glutaredoxin | Escherichia phage SUSP1 | 2e-52 | 100% | 98%(100%) | [YP_009199315.1](https://www.ncbi.nlm.nih.gov/protein/YP_009199315.1?report=genbank&log$=prottop&blast_rank=2&RID=S2056S8X016) |  |
| 92^a^+ | 64045 | 64245 | 66 | 7.589 | 7.14 | putative membrane protein | Escherichia phage mio | 4e-20 | 100% | 62%(78%) | [QIG66592.1](https://www.ncbi.nlm.nih.gov/protein/QIG66592.1?report=genbank&log$=prottop&blast_rank=13&RID=S205JMAC013) |  |
| 93^a^+ | 64295 | 66436 | 713 | 80.581 | 6.27 | anaerobic ribonucleoside-triphosphate reductase | Escherichia coli | 0.0 | 100% | 99%(99%) | [EFG4626532.1](https://www.ncbi.nlm.nih.gov/protein/EFG4626532.1?report=genbank&log$=prottop&blast_rank=1&RID=S205Y0JN013) |  |
| 94^a^+ | 66500 | 66682 | 60 | 7.032 | 5.80 | hypothetical protein CPT_Michonne_gp015 | Citrobacter phage Michonne | 4e-35 | 100% | 100%(100%) | YP_009177262.1 |  |
| 95^a^+ | 66679 | 66942 | 87 | 10.466 | 4.13 | hypothetical protein CPT_Michonne_gp014 | Citrobacter phage Michonne | 5e-56 | 100% | 98%(98%) | [YP_009177261.1](https://www.ncbi.nlm.nih.gov/protein/YP_009177261.1?report=genbank&log$=prottop&blast_rank=1&RID=S206RM48013) |  |
| 96^a^+ | 66939 | 67042 | 33 | 3.624 | 9.44 | NO |  |  |  |  |  |  |
| 97^a^+ | 67033 | 67425 | 130 | 14.912 | 4.44 | tail tube protein | Shigella phage vB_SflM_004 | 2e-41 | 97% | 52%(72%) | [AZV01433.1](https://www.ncbi.nlm.nih.gov/protein/AZV01433.1?report=genbank&log$=prottop&blast_rank=22&RID=S27EPB54016) |  |
| 98^a^+ | 67425 | 67910 | 161 | 18.676 | 7.29 | anaerobic NTP reductase small subunit | Citrobacter phage Michonne | 2e-115 | 100% | 98%(99%) | [YP_009177259.1](https://www.ncbi.nlm.nih.gov/protein/YP_009177259.1?report=genbank&log$=protalign&blast_rank=1&RID=S27FCJGY016) |  |
| 99^a^+ | 68209 | 68466 | 85 | 8.887 | 11.85 | hypothetical protein CPT_Moogle11 | Citrobacter phage Moogle | 7e-51 | 100% | 100%(100%) | [YP_009145654.1](https://www.ncbi.nlm.nih.gov/protein/YP_009145654.1?report=genbank&log$=prottop&blast_rank=1&RID=S27FTZHZ013) |  |
| 100^a^+ | 68478 | 68789 | 103 | 11.981 | 8.79 | putative HNH endonuclease | Shigella phage KPS64 | 4e-64 | 100% | 91%(97%) | [QBP32771.1](https://www.ncbi.nlm.nih.gov/protein/QBP32771.1?report=genbank&log$=prottop&blast_rank=1&RID=S27HWVVR016) |  |
| 101^a^+ | 68842 | 69357 | 171 | 20.285 | 8.74 | hypothetical protein AVU07_agp073 | Escherichia phage SUSP1 | 2e-123 | 100% | 97%(98%) | [YP_009199324.1](https://www.ncbi.nlm.nih.gov/protein/YP_009199324.1?report=genbank&log$=prottop&blast_rank=1&RID=S27J8BN6013) |  |
| 102^a^+ | 69305 | 69628 | 92 | 10.422 | 5.25 | hypothetical protein AVU07_agp074 | Escherichia phage SUSP1 | 1e-62 | 100% | 99%(100%) | [YP_009199325.1](https://www.ncbi.nlm.nih.gov/protein/YP_009199325.1?report=genbank&log$=prottop&blast_rank=1&RID=S27K00DM013) |  |
| 103^a^+ | 69639 | 70520 | 293 | 32.261 | 4.99 | ribose-phosphate pyrophosphokinase | Citrobacter phage Michonne | 0.0 | 100% | 96%(98%) | [YP_009177253.1](https://www.ncbi.nlm.nih.gov/protein/YP_009177253.1?report=genbank&log$=protalign&blast_rank=2&RID=S27KDERZ016) |  |
| 104^a^+ | 70565 | 72382 | 605 | 67.978 | 5.04 | nicotinate phosphoribosyltransferase | Escherichia coli | 0.0 | 100% | 97%(98%) | [EFG4626543.1](https://www.ncbi.nlm.nih.gov/protein/EFG4626543.1?report=genbank&log$=prottop&blast_rank=2&RID=S27MC2AH016) |  |
| 105^a^+ | 72436 | 72651 | 72651 | 8.721 | 9.49 | DNA polymerase | Shigella phage vB_SflM_004 | 4e-40 | 100% | 92%(94%) | [AZV01424.1](https://www.ncbi.nlm.nih.gov/protein/AZV01424.1?report=genbank&log$=prottop&blast_rank=6&RID=S27MWACU016) |  |
| 106^a^+ | 72648 | 72983 | 111 | 12.799 | 6.61 | hypothetical protein | Escherichia coli | 9e-75 | 100% | 98%(100%) | [EFG4626545.1](https://www.ncbi.nlm.nih.gov/protein/EFG4626545.1?report=genbank&log$=prottop&blast_rank=1&RID=S27N8077013) |  |
| 107^a^+ | 72965 | 73144 | 59 | 6.392 | 5.74 | hypothetical protein AVU07_agp073 | Escherichia phage SUSP1 | 2e-32 | 100% | 97%(98%) | YP_009199331.1 |  |
| 108^a^+ | 73207 | 75579 | 790 | 90.338 | 7.04 | rIIa | Citrobacter phage Moogle | 0.0 | 100% | 99%(99%) | [YP_009145644.1](https://www.ncbi.nlm.nih.gov/protein/YP_009145644.1?report=genbank&log$=prottop&blast_rank=1&RID=S288KKHT013) |  |
| 109^a^+ | 75659 | 76780 | 373 | 41.712 | 9.14 | rIIb | Citrobacter phage Moogle | 0.0 | 100% | 99%(99%) | [YP_009199333.1](https://www.ncbi.nlm.nih.gov/protein/YP_009199333.1?report=genbank&log$=prottop&blast_rank=2&RID=S288YANM016) |  |
| 110^a^+ | 76881 | 77429 | 182 | 20.813 | 9.82 | putative lysin | Shigella phage Silverhawkium | 4e-119 | 100% | 89%(95%) | [QBP33221.1](https://www.ncbi.nlm.nih.gov/protein/QBP33221.1?report=genbank&log$=protalign&blast_rank=7&RID=S289ASDK016) |  |
| 111^a^+ | 77407 | 78102 | 231 | 26.003 | 5.39 | polynucleotide kinase  hypothetical protein | Shigella phage Sf13 | 7e-166 | 100% | 97%(98%) | YP_009612678.1 |  |
| 112^a^+ | 78114 | 78578 | 154 | 16.297 | 9.68 | hypothetical protein FDI44_gp083 | Shigella phage Sf13 | 6e-106 | 100% | 99%(100%) | [YP_009612677.1](https://www.ncbi.nlm.nih.gov/protein/YP_009612677.1?report=genbank&log$=prottop&blast_rank=1&RID=S28AH8VE013) |  |
| 113^a^+ | 78631 | 78978 | 115 | 13.199 | 8.38 | spanin | Escherichia phage SUSP1 | 4e-79 | 100% | 100%(100%) | [EGE3642132.1](https://www.ncbi.nlm.nih.gov/protein/EGE3642132.1?report=genbank&log$=prottop&blast_rank=1&RID=S28AV8VF013) |  |
| 114^b^+ | 78944 | 79141 | 65 | 7.507 | 5.19 | o-spanin | Escherichia phage SUSP1 | 5e-39 | 100% | 98%(100%) | YP_009305400.1 |  |
| 115^a^+ | 79138 | 79407 | 89 | 10.555 | 6.33 | AVU07_agp088  hypothetical protein | Escherichia phage SUSP1 | 3e-61 | 100% | 100%(100%) | YP_009199339.1 |  |
| 116^a^+ | 79411 | 79863 | 150 | 17.129 | 4.60 | CPT_Michonne_gp093  hypothetical protein | Citrobacter phage Michonne | 1e-60 | 100% | 99%(99%) | [YP_009177384.1](https://www.ncbi.nlm.nih.gov/protein/YP_009177384.1?report=genbank&log$=prottop&blast_rank=2&RID=S28BJY1A013) |  |
| 117^a^+ | 79873 | 80199 | 108 | 12.448 | 4.83 | tail assembly protein | Shigella flexneri | 9e-42 | 100% | 58%(81%) | [QHJ72555.1](https://www.ncbi.nlm.nih.gov/protein/QHJ72555.1?report=genbank&log$=prottop&blast_rank=20&RID=S2BATZ7Z016) |  |
| 118^a^+ | 80391 | 80591 | 66 | 7.375 | 4.40 | CPT_Michonne_gp096  macro | Citrobacter phage Michonne | 1e-41 | 100% | 97%(100%) | [YP_009177380.1](https://www.ncbi.nlm.nih.gov/protein/YP_009177380.1?report=genbank&log$=prottop&blast_rank=1&RID=S2BBX4AT013) |  |
| 119^a^+ | 80593 | 81387 | 264 | 29.333 | 8.10 | domain-containing protein | Escherichia coli | 0.0 | 100% | 98%(99%) | [EFG4626556.1](https://www.ncbi.nlm.nih.gov/protein/EFG4626556.1?report=genbank&log$=prottop&blast_rank=1&RID=S2BD17D9013) |  |
| 120^a^+ | 81859 | 82101 | 68 | 8.486 | 8.85 | hypothetical protein | Citrobacter freundii | 6e-42 | 100% | 100%(100%) | [HAU5726791.1](https://www.ncbi.nlm.nih.gov/protein/HAU5726791.1?report=genbank&log$=prottop&blast_rank=1&RID=S2BKSY7C013) |  |
| 121^a^+ | 82115 | 82276 | 53 | 6.002 | 8.71 | Sf18_gp72  hypothetical protein | Shigella phage Sf18 | 8e-25 | 100% | 92%(94%) | ATE86346.1 |  |
| 122^a^+ | 82365 | 82745 | 126 | 14.288 | 9.13 | hypothetical protein | Escherichia coli | 3e-87 | 100% | 100%(100%) | EFG4626558.1 |  |
| 123^a^+ | 82832 | 83089 | 85 | 9.343 | 10.30 | CPT_Michonne_gp102  hypothetical protein | Citrobacter phage Michonne | 3e-49 | 100% | 91%(96%) | YP_009177374.1 |  |
| 124^a^+ | 83179 | 83691 | 170 | 19.521 | 4.51 | hypothetical protein | Escherichia coli | 1e-110 | 100% | 90%(95%) | EFG4626560.1 |  |
| 125^a^+ | 83782 | 84069 | 95 | 10.997 | 9.65 | CHB7_gp122  tail sheath monomer | Enterobacteria phage CHB7 | 2e-43 | 100% | 83%(91%) | [QBP33073.1](https://www.ncbi.nlm.nih.gov/protein/QBP33073.1?report=genbank&log$=protalign&blast_rank=4&RID=S2C05TNS016) |  |
| 126^a^+ | 84074 | 84397 | 107 | 12.215 | 7.14 | hypothetical protein mistaenkt_12 | Escherichia phage mistaenkt | 2e-45 | 100% | 64%(84%) | [QHR67900.1](https://www.ncbi.nlm.nih.gov/protein/QHR67900.1?report=genbank&log$=protalign&blast_rank=1&RID=S2HK4E2V016) |  |
| 127^a^+ | 84494 | 84766 | 90 | 10.341 | 9.61 | AVU07_agp102  hypothetical protein | Escherichia phage SUSP1 | 3e-57 | 100% | 98%(98%) | YP_009199353.1 |  |
| 128^a^+ | 84853 | 85254 | 133 | 15.031 | 4.58 | hypothetical protein | Shigella flexneri | 6e-85 | 100% | 91%(97%) | [EFV7170025.1](https://www.ncbi.nlm.nih.gov/protein/EFV7170025.1?report=genbank&log$=protalign&blast_rank=2&RID=S2HN3H18013) |  |
| 129^a^+ | 85320 | 85562 | 80 | 9.251 | 9.58 | CHB7_gp118  hypothetical protein | Enterobacteria phage CHB7 | 2e-45 | 100% | 93%(93%) | QBP33070.1 |  |
| 130^a^+ | 85656 | 86213 | 185 | 21.123 | 3.87 | CPT_Moogle108  hypothetical protein | Citrobacter phage Moogle | 6e-132 | 100% | 97%(98%) | [YP_009145751.1](https://www.ncbi.nlm.nih.gov/protein/YP_009145751.1?report=genbank&log$=protalign&blast_rank=1&RID=S2HNTMNM013) |  |
| 131^a^+ | 86277 | 86507 | 76 | 8.756 | 3.91 | CPT_Moogle107  hypothetical protein | Citrobacter phage Moogle | 2e-46 | 100% | 97%(100%) | [YP_009145750.1](https://www.ncbi.nlm.nih.gov/protein/YP_009145750.1?report=genbank&log$=protalign&blast_rank=1&RID=S2HPAF88013) |  |
| 132^a^+ | 86589 | 86930 | 113 | 13.327 | 7.23 | AVU07_agp108  hypothetical protein | Escherichia phage SUSP1 | 1e-77 | 100% | 96%(99%) | YP_009199359.1 |  |
| 133^a^+ | 87131 | 87658 | 175 | 20.240 | 8.06 | CPT_Moogle105  hypothetical protein | Citrobacter phage Moogle | 1e-126 | 100% | 98%(100%) | YP_009145748.1 |  |
| 134+ | 87717 | 87980 | 87 | 9.899 | 4.52 | hypothetical protein | Citrobacter phage Moogle | 3e-55 | 100% | 98%(97%) | [YP_009145747.1](https://www.ncbi.nlm.nih.gov/protein/YP_009145747.1?report=genbank&log$=protalign&blast_rank=1&RID=S2HRMZ7R013) |  |
| 135^a^+ | 88045 | 88293 | 82 | 9.545 | 4.31 | Putative membrane protein | Shigella phage Sf18 | 1e-13 | 100% | 39.0 (32) | ATE86363.1 |  |

a, ATG start codon; b, GTG start codon; c, TTG start codon.

+, right orientation; -, left orientation; —, no homology to the known sequences.
